# Macrophage beta2-adrenergic receptor is dispensable for the adipose tissue inflammation and function

**DOI:** 10.1016/j.molmet.2021.101220

**Published:** 2021-03-25

**Authors:** Kasparas Petkevicius, Guillaume Bidault, Sam Virtue, Stephen A. Newland, Martin Dale, Aurelien Dugourd, Julio Saez-Rodriguez, Ziad Mallat, Antonio Vidal-Puig

**Affiliations:** 1 University of Cambridge Metabolic Research Laboratories, Institute of Metabolic Science, MDU MRC, Cambridge, United Kingdom; 2 Division of Cardiovascular Medicine, University of Cambridge, Cambridge, United Kingdom; 3 Joint Research Centre for Computational Biomedicine, Faculty of Medicine, RWTH Aachen University, Aachen, Germany; 4 Institute for Computational Biomedicine, Heidelberg University, Faculty of Medicine & Heidelberg University Hospital, Heidelberg, Germany; 5 Wellcome Trust Sanger Institute, Hinxton, United Kingdom

**Keywords:** Immunometabolism, Neuroimmune, Sympathetic, Norepinephrine, adrb2, Atherosclerosis

## Abstract

**Objective:**

Neuroimmune interactions between the sympathetic nervous system (SNS) and macrophages are required for the homeostasis of multiple tissues, including the adipose tissue. It has been proposed that the SNS maintains adipose tissue macrophages (ATMs) in an anti-inflammatory state via direct norepinephrine (NE) signaling to macrophages. This study aimed to investigate the physiological importance of this paradigm by utilizing a mouse model in which the adrenergic signaling from the SNS to macrophages, but not to other adipose tissue cells, was disrupted.

**Methods:**

We generated a macrophage-specific B2AR knockout mouse (*Adrb2*^ΔLyz2^) by crossing *Adrb2*^fl/fl^ and *Lyz2*^Cre/+^ mice. We have previously shown that macrophages isolated from *Adrb2*^ΔLyz2^ animals do not respond to NE stimulation *in vitro*. Herein we performed a metabolic phenotyping of *Adrb2*^ΔLyz2^ mice on either chow or high-fat diet (HFD). We also assessed the adipose tissue function of *Adrb2*^ΔLyz2^ animals during fasting and cold exposure. Finally, we transplanted *Adrb2*^ΔLyz2^ bone marrow to low-density lipoprotein receptor (LDLR) knockout mice and investigated the development of atherosclerosis during Western diet feeding.

**Results:**

We demonstrated that SNS-associated ATMs have a transcriptional profile indicative of activated beta-2 adrenergic receptor (B2AR), the main adrenergic receptor isoform in myeloid cells. However, *Adrb2*^ΔLyz2^ mice have unaltered energy balance on a chow or HFD. Furthermore, *Adrb2*^ΔLyz2^ mice show similar levels of adipose tissue inflammation and function during feeding, fasting, or cold exposure, and develop insulin resistance during HFD at the same rate as controls. Finally, macrophage-specific B2AR deletion does not affect the development of atherosclerosis on an LDL receptor-null genetic background.

**Conclusions:**

Overall, our data suggest that the SNS does not directly modulate the phenotype of adipose tissue macrophages in either lean mice or mouse models of cardiometabolic disease. Instead, sympathetic nerve activity exerts an indirect effect on adipose tissue macrophages through the modulation of adipocyte function.

## Highlights

• Sympathetic nerve-associated macrophages in white adipose tissues exhibit a transcriptional signature of B2AR activation.
• Macrophage-specific B2AR knockout does not affect the phenotype of adipose tissue macrophages.
• Macrophage B2AR signaling does not affect adipose tissue function during fasting or cold exposure.
• Macrophage B2AR is dispensable for the development of insulin resistance and atherosclerosis in obesity.

## Graphical abstract

**Figure:**
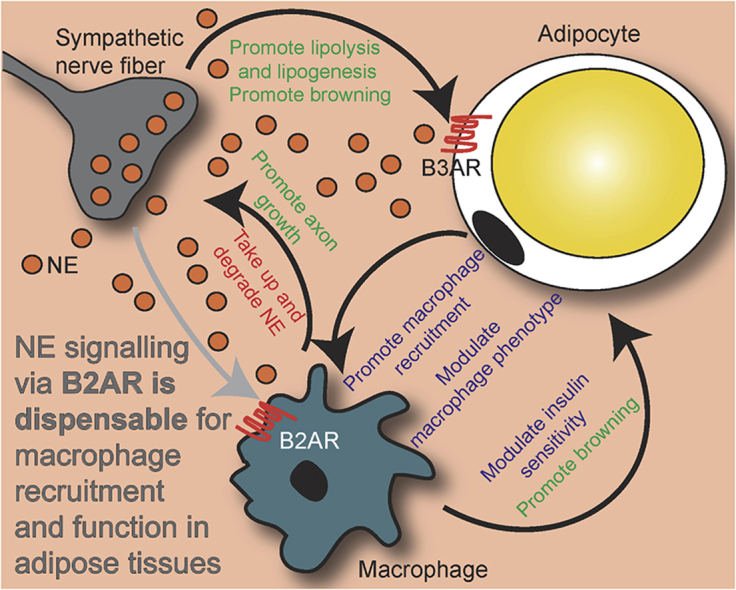

## 1 Introduction

Obesity-associated cardiometabolic diseases are among the most prevailing causes of death worldwide. White adipose tissue (WAT) function is one of the key determinants of metabolic disease development [1]. When WAT function is impaired, other tissues accumulate excess lipids, leading to the development of multiple disorders, such as insulin resistance and atherosclerosis [1]. Two of the main physiological systems that regulate adipose tissue function are the sympathetic nervous system (SNS) [2] and the immune system [3]. In a healthy state, sympathetic nerves innervating adipose tissues secrete norepinephrine (NE) to stimulate adipocyte lipolysis, mitochondrial biogenesis and adipose tissue remodeling [2]. Similarly, WAT-resident immune cells, in particular adipose tissue macrophages (ATMs), maintain WAT homeostasis by remodeling extracellular matrix and clearing apoptotic cells [3]. In obesity and ageing, NE signaling in WAT is attenuated, leading to an impaired control of lipolysis and energy expenditure [2,4]. Furthermore, obesity is associated with WAT inflammation, characterized by an increased ATM number and a phenotypic switch from an anti-inflammatory (M2) to a pro-inflammatory (M1) polarization state [3]. M1-polarized ATMs then secrete pro-inflammatory cytokines that cause adipocyte insulin resistance [3]. The pathophysiological mechanisms responsible for the obesity-related dysregulation of both the SNS and the immune system, that in turn leads to impaired adipocyte function and the development of insulin resistance, are presently not understood well enough to be successfully targeted therapeutically.

While the interplay between the tissue sympathetic innervation and macrophages has been observed in multiple physiological and pathological settings, it is arguably best characterized in the intestine. Intestinal muscle-resident macrophages (muscularis macrophages, or MMs) physically interact with intestinal neurons and produce the signaling factor bone morphogenic protein 2 (BMP2) that modulates neuronal function, thus affecting muscle contractility and intestinal motility [5]. Simultaneously, intestinal neurons secrete colony stimulatory factor 1 (CSF1) that is required for the survival of the MM [5]. Furthermore, enteric bacterial infection increases SNS outflow to the intestinal muscle and engages beta2-adrenergic receptors (B2ARs) on MMs via direct NE signaling [6]. B2AR activation promotes an anti-inflammatory M2 phenotype in macrophages, and therefore, loss of macrophage B2AR signaling leads to unresolved intestinal inflammation that culminates in neuronal death [7]. Equally, sympathetic denervation of the intestine causes colitis [8]. Altogether, interactions between sympathetic nerves and macrophages are crucial for intestinal homeostasis.

Physical interactions between sympathetic nerves and macrophages have recently been observed in mouse and human adipose tissues [[9], [10], [11]]. ATMs have been shown to secrete neurotrophic factors that promote sympathetic nerve axon branching in brown adipose tissue (BAT) and subcutaneous WAT (scWAT) [10,12]. Sympathetic nerve-associated macrophages also express the molecular machinery required to take up and degrade NE [9,11]. Ageing leads to an expansion of the NE-degrading ATM population [11], while obesity induces a more pro-inflammatory phenotype in the ATMs that interact with sympathetic nerves, compared to the overall scWAT macrophage population [9]. Genetic or pharmacological inhibition of the NE degradation pathway in macrophages increases SNS tone in adipose tissues and reverts the adipocyte lipolytic and thermogenic impairments caused by ageing or obesity [9,11]. It has also been demonstrated that NE-dependent SNS signaling to macrophage B2ARs maintains ATMs in an anti-inflammatory M2 polarization state [13,14]. In line with an anti-inflammatory role of B2AR signaling in macrophages, sympathetic denervation of BAT or WAT therefore leads to ATM M1 polarization and increased adipose tissue inflammation [13]. Furthermore, leptin administration to the obese leptin-deficient mouse model has been shown to rapidly stimulate SNS outflow to WAT, elevate WAT NE levels, and induce an anti-inflammatory phenotype in ATMs [14]. Overall, the current model of WAT biology indicates a bidirectional relationship between sympathetic nerves and ATMs [2,15], which is somewhat analogous to the neuroimmune interactions between MMs and intestinal neurons.

Herein we aimed to determine the physiological importance of adrenergic signaling to ATMs. In accordance with a previous report [13], our findings indicate that B2AR is the main adrenergic receptor in ATMs, and that sympathetic nerve-associated macrophages in scWAT have a transcriptional profile indicative of B2AR activation. However, macrophage-specific B2AR deletion does not affect the ATM phenotype, WAT function, or systemic metabolism in states where adipose tissue function is challenged, namely fasting, cold exposure, and obesity. Our data therefore argue against the view that the SNS can directly modulate ATM function via NE signaling.

## 2 Materials and methods

### 2.1 Mice and diets

This research conformed to the Animals (Scientific Procedures) Act 1986 Amendment Regulations 2012 following ethical review by the University of Cambridge Animal Welfare and Ethical Review Body (AWERB). Unless otherwise indicated, mice were housed 3–5 per-cage in a temperature-controlled room (21 °C) with a 12-h light/dark cycle, with ‘lights on’ corresponding to 6 am. The animals had ad libitum access to food and water. Standard chow diet was purchased from Safe diets (DS-105), high-fat diet (HFD) from Research diets (D12492), and Western diet (WD) from Dietex (21% fat, 0.15% cholesterol). For cold exposure studies, cages were transferred to a temperature controlled room set at 4 °C and relative humidity at 50%, and body weight and rectal temperature measurements were performed within that room.

Macrophage-specific *Adrb2* knockout mice were generated by crossing a mouse model containing loxP sequences surrounding *Adrb2* alleles (*Adrb2*^fl/fl^) to the *Lyz2*^+/Cre^ mouse. The *Adrb2*^fl/fl^ mouse was generated by Prof. Florent Elefteriou and Prof. Gerard Karsenty as described [16] and was kindly gifted to us by Prof. Gerard Karsenty on a pure C57Bl/6 J genetic background. All experimental macrophage-specific knockout mice were produced by crossing *Lyz2*^+/+^ with *Lyz2*^+/Cre^ animals on a floxed/floxed background, yielding a 1:1 Mendelian ratio of control (floxed/floxed *Lyz2*^+/+^) to knockout (floxed/floxed *Lyz2*^+/Cre^) offspring.

### 2.2 Bone marrow transplant

*Ldlr*^*−/−*^ recipient mice (eight-week-old females, Jackson Labs 002207) were given antibiotics in drinking water (Baytril) overnight. The next day, mice were subjected to two doses of 5.5 Gy irradiation (separated by 4 h), followed by the intravenous injection with 1 × 10^7^ sex-matched donor (*Adrb2*^fl/fl^ or *Adrb2*^fl/fl^ *Lyz2*^+/Cre^) bone marrow cells. Mice were maintained on Baytril for 4 weeks before being placed on WD for 12 weeks.

### 2.3 Indirect calorimetry

Mice were single-housed one week before the experiment to adjust. Energy expenditure (EE) was calculated from data gathered from single-housed mice fed ad libitum by a customized monitoring system (Creative Scientific UK). The monitoring system calculated oxygen and carbon dioxide concentrations at 18-min intervals, with flow rates set to 400 mL/min. EE was calculated from oxygen consumption and carbon dioxide production measured over a 48-h period using the modified Weir equation (EE J/min = 15.818 × VO_2_ (mL/min) + 5.176 × VCO_2_ (mL/min)).

### 2.4 Food intake assessment

Mice were single-housed one week before the experiment to adjust. Daily food intake and fecal excretion were assessed by single-housing mice in cages containing liners and manually weighing food pellets and crumbs (food intake) for two consecutive weeks. Feces were weighed during the second week only.

### 2.5 Blood sampling and serum biochemistry

Blood was sampled from tail veins. Terminal blood samples were obtained by cardiac puncture and collected into blood collection tubes (0103, Vetlab). Blood samples for insulin, FFA and TG measurements were collected into glass capillary tubes. Blood samples were kept on ice before centrifuging at >10,000 *g* for 5 min. Serum was then collected and stored at −80 °C for subsequent analyses.

Blood glucose was measured using AlphaTRAK 2 glucose meters and strips (Zoetis). Serum insulin was measured using electrochemical luminescence immunoassay (K152BZC, MesoScale Discovery). Serum TG and FFA was measured enzymatically using commercially available kits (TG- Siemens Healthcare and FFA- 1138175001, Roche).

### 2.6 Glucose tolerance tests

Mice were fasted for 16 h from 4 pm to 8 am. Mice were single-housed at least 1 h prior to being injected intraperitoneally with 1 mg/kg glucose. Blood was sampled at 0, 10, 20, 30, 60, 90, and 120 min after the injection.

### 2.7 Insulin tolerance tests

Mice were fasted for 6 h from 8 am to 2 pm. Mice were single-housed at least 1 h prior to being injected intraperitoneally with 0.75 IU/kg of human insulin (ActRapid). An insulin dose of 1 IU/kg was used for HFD-fed mice. Blood was sampled at 0, 10, 20, 30, 60, 90, and 120 min after the injection.

### 2.8 Hyperinsulinemic-euglycemic clamp

Hyperinsulinemic-euglycemic clamp experiment was performed as described in detail [17]. Briefly, mice were anaesthetized and maintained at 37 °C through the procedure. Tail veins were catheterized and mice underwent a basal perfusion with [3-^3^H]-glucose. A bolus of insulin was then administered to initiate hyperinsulinemia, and hyperinsulinemic infusate continued to be administered for at least 90 min. Immediately after initiation of hyperinsulinemia, glucose solution was infused at a variable rate in order to clamp glucose at the basal level. Blood glucose was measured every 5 min for 90 min and glucose infusion rate (GIR) was adjusted until mice had stable blood glucose values that were +/− 0.5 mM of their initial values. Serum samples were collected for the determination of specific activity at the end of basal and hyperinsulinemic states.

### 2.9 Adipose tissue cell fractionation

Adipose tissues were removed after sacrifice, chopped thoroughly and re-suspended in 10 mL digestion solution containing 7 mL Hanks’ Balanced Salt Solution (HBSS, H9269, Sigma), 0.23 g bovine serum albumin (BSA, A9418, Sigma), and 20 mg collagenase type II (C6885, Sigma) filtered through a 0.22-μm membrane. The digestion was performed at 37 °C for 20 min (eWAT pads) or 30 min (scWAT pads), with shaking at 100 rpm. The digestion mixture was then passed through a 100 μm cell strainer (352,360, Falcon) into a fresh tube and incubated at room temperature for 10 min, allowing the adipocyte fraction to layer on the surface. The adipocyte fraction was collected by pipetting and frozen for subsequent analysis. The remaining solution containing adipose tissue stromal vascular fraction (SVF) was centrifuged at 400 g, 4 °C for 5 min and pellet was re-suspended in 1 mL of pre-cooled (at 4 °C) selection buffer (2 mM EDTA, 0.5% bovine serum albumin in phosphate buffered saline [PBS]). Total cell number was determined by manual counting. The cell suspension was centrifuged at 400 g, 4 °C for 5 min and the pellet was re-suspended in 90 μL of selection buffer and 10 μL of CD11b micro-beads (130,049,601, Miltenyi Biotec) per 10^7^ cells. Cell suspension was mixed and incubated for 15 min at 4 °C. Cells were then washed by adding 3 mL of selection buffer and centrifuged at 400 g at 4 °C for 5 min. The pellet was re-suspended in 500 μL of selection buffer and loaded onto a MACS LS column (130-042-401, Miltenyi Biotec) placed in the magnetic field of a MACS separator (Miltenyi Biotec) and flow-through was collected in a fresh tube. The column was then washed thrice with 3 mL of selection buffer. The total effluent contained unlabeled cells and corresponded to the adipose tissue CD11b-negative cell fraction. The LS column was then removed from the MACS separator and placed in a fresh collection tube. The labelled cells were eluted using 5 mL of selection buffer and corresponded to adipose tissue macrophage (ATM) fraction. Both CD11b-positive and CD11b-negative fractions were centrifuged at 400 *g*, 4 °C for 5 min, and pellets were frozen and stored at −80 °C for subsequent analysis.

Sympathetic nerve-associated macrophages were isolated from scWAT by Pirzgalska et al., as described in detail in the original publication [9]. Briefly, the authors manually isolated sympathetic nerve fibers innervating scWAT and digested them with hyalurodinase at 37 °C for 30 min, then with collagenase at 37 °C for further 15 min [9]. From the resulting cell suspension, sympathetic nerve-associated macrophages were sorted using fluorescence-activated cell sorting as live CD45 and F4/80 double-positive cells [9].

### 2.10 Bone marrow-derived macrophage culture and stimulations

Bone marrow-derived macrophages (BMDMs) were differentiated from femur and tibia bone marrow cells using L929 cell-conditioned medium for 7 day as described previously in detail [18]. On day 7, BMDMs were replated into experimental plates and stimulated the following day as described [18].

### 2.11 Flow cytometry

SVF from digested adipose tissues was collected and kept in FACS buffer (PBS, 1 mM EDTA, 3% HI-FBS) on ice. Spleens were dissociated into cells by passing them through the cell strainer with a syringe plunger into the FACS buffer. After red blood cell lysis (420,301, Biolegend), cell numbers were determined using Countess II (Thermo Fisher). Cells were stained with LIVE/DEAD (L34964, Thermo Fisher) and non-specific binding was blocked with 5 μg/mL anti-CD16/32 (Biolegend). Cell surfaces were then stained with anti-CD45 (564,279, BD), anti-CD11b (564,443, BD), anti-Siglec-F (562,757, BD), anti-F4/80 (123,116, BioLegend), anti-CD301 (145,704, BioLegend), anti-CD206 (141,723, BioLegend), anti-CD11c (117,336, BioLegend), anti-CD19 (562,335, BD), anti-CD3e (564379, BD), anti-Ly6G (127,608, BioLegend), anti-Ly6C (128,046, BioLegend), anti-CD4 (100,511, BioLegend), anti-CD8 (100,713, BioLegend). For the intracellular Foxp3 staining, cells were fixed and permeabilized using the Foxp3 staining kit according to the manufacturer's protocol (00-5523-00, Thermo Fisher) and stained for 1 h at 4 °C with the FITC-FoxP3 antibody (FJK-16s, Thermo Fisher).

Myeloid cells were gated within the live single cell population as follows: macrophages: CD45+/CD11b+/Ly6G-/SiglecF-/F4/80+; monocytes: CD45+/CD11b+/Ly6G-/SiglecF-/F4/80-/Ly6C+/SSC_low_; eosinophils: CD45+/CD11b+/Ly6G-/SiglecF+; and neutrophils: CD45+/CD11b+/Ly6G+. Lymphoid cells were gated as follows: B cells: CD45+/CD19+; CD4^+^ T cells: CD45+/CD19-/CD3+/CD4+; Tregs were FoxP3+ within CD4^+^ T cell population; CD8^+^ T cells: CD45+/CD19-/CD3+/CD8+. Data were acquired on LSRFortessa (BD Biosciences) using FACS Diva software and analyzed with TreeStar FlowJo (Version vX0.7).

### 2.12 Cytokine measurements

Serum cytokine levels were determined in undiluted serum samples using a customized mouse U-PLEX assay platform (Meso Scale Diagnostics) according to the manufacturer's protocol.

### 2.13 Extent of atherosclerotic lesions

Tissues were fixed in 1% paraformaldehyde overnight before washing with PBS. Quantification of atherosclerosis was performed using Oil Red O staining as previously described [19]. Briefly, en face whole mount staining was performed on cleaned aorta using 0.5% solution of Oil Red O (O0625, Sigma) in 60% isopropyl alcohol (working solution). Aortas were then rinsed in water followed by 2 washes in 70% isopropyl alcohol before immersion in Oil Red O working solution for 45 min. This was followed by 2 washes in 70% isopropyl alcohol and 3 additional washes in water. Lesion size was quantified using Fiji [20].

### 2.14 RNA isolation

RNA from tissues and adipocyte fractions was isolated using phenol-chloroform extraction method with Stat-60 reagent as described [18]. RNA from BMDMs and CD11b-positive cell fractions was isolated using RNeasy Plus Mini kit (74,106, Qiagen) and RNeasy Plus Micro kit (74,004, Qiagen), respectively, following the manufacturer's protocol.

### 2.15 Quantitative real-time polymerase chain reaction (qRT-PCR)

Complementary DNA (cDNA) was generated from 500 ng RNA using Promega reagents as described [18] and diluted 75-fold in RNAse-free water for subsequent analysis. qRT-PCR was performed in a 13 μL reaction with 5 μL of diluted cDNA, 6.5 μL of 2x TaqMan or SYBR Green reagent (Applied Biosystems), 1.3 μL of 3 mM forward and reverse primer mix (including 1.5 mM of probe for TaqMan reactions) and 0.2 μL of RNAse-free water according to the default manufacturer's protocol (Applied Biosystems). Primer sequences are listed in Supplementary Table S1. Reactions were run in duplicate for each sample and quantified using the ABI Prism 7900 sequence detection system (Applied Biosystems). Duplicates were checked for reproducibility, and then averaged; ‘no reverse transcriptase’ controls were included to check for genomic DNA contamination, and ‘no template’ controls were included to check for the formation of primer dimers. Product specificity was determined using a dissociation curve for SYBR green reactions. A standard curve generated from a pool of all cDNA samples was used for quantification. The expression of genes of interest was normalized using the BestKeeper method to the geometric average of 3–4 housekeeping genes (*18s*, *Actb*, *36b4* and *Tbp*), and data were expressed as arbitrary units or normalized to the average of control group.

### 2.16 Statistical analysis and graphical representation of data

All data from experiments are represented as a mean, with error bars showing standard error of the mean and the number of replicates stated in legend. Some data are represented as a fold-change, and it is stated in legend to what value the data represented was normalized to generate the fold-change. Statistical tests used are also stated in the legend. A student's *t*-test was used to compare two groups; one-way analysis of variance (ANOVA) was used to compare more than two groups, followed by Bonferonni's post-hoc test. Where more than one factor influenced the variable being measured, 2-way ANOVA was used to test for a significant effect of each factor as well as an interaction between factors. To compare the differences between the intercept values of the linear regression lines of two groups, analysis of co-variance (ANCOVA) was used.

All statistical tests were performed and graphs were generated using GraphPad Prism v8 software. In order to assess similarities between RNA sequencing datasets, we used GSEA to see if the DEG by B2AR agonist in BMDMs were significantly enriched in the top 500 DEG of a tissue macrophage dataset, and vice-versa. Graphs and figures were edited for presentation using Adobe Illustrator CC 2015 software.

## 3 Results

### 3.1 The transcriptional profile of SNS-associated adipose tissue macrophages mimics acute B2AR activation

**Figure 1:**
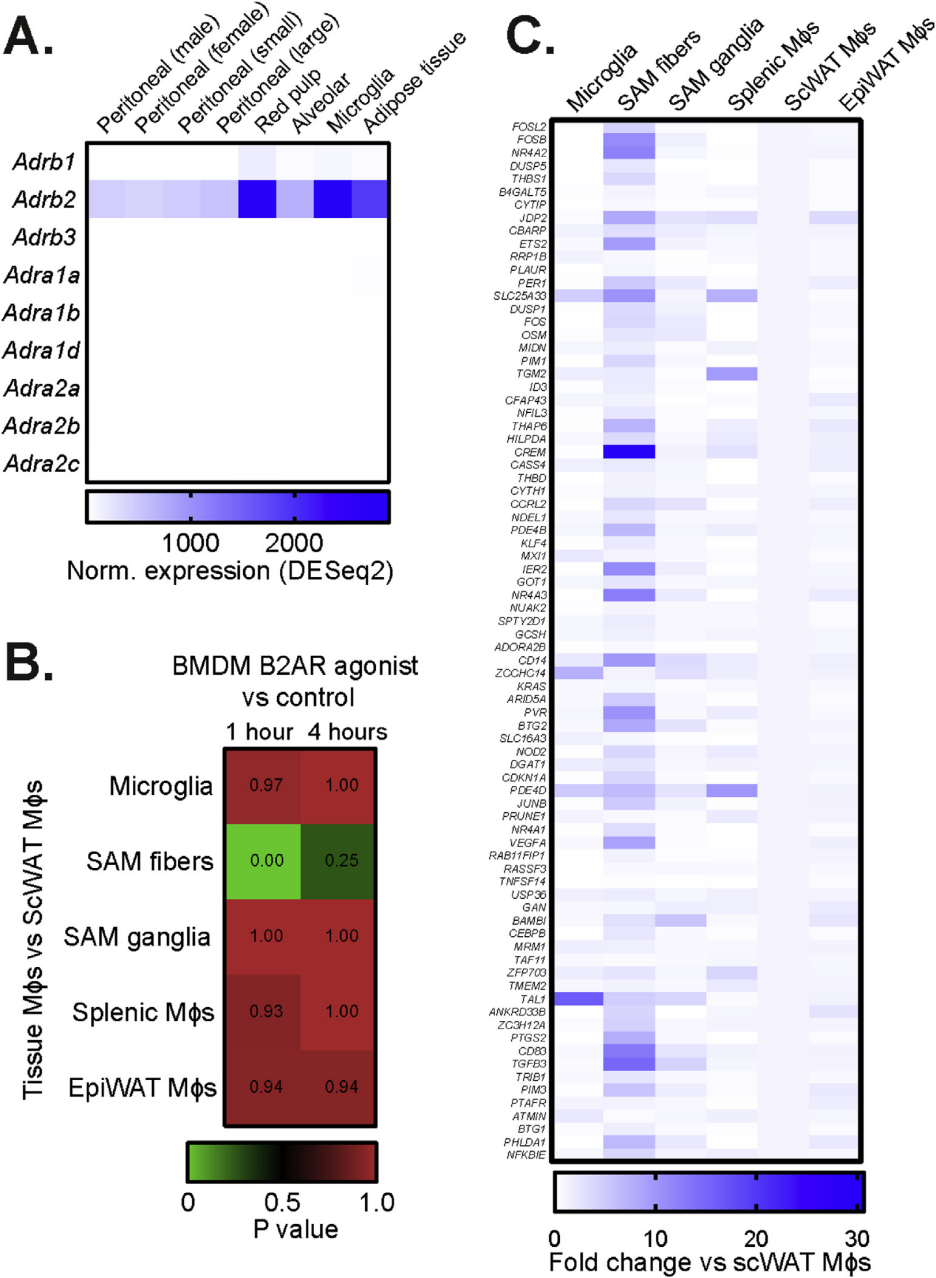
**Sympathetic nerve-associated macrophages in scWAT exhibit a transcriptional signature of B2AR activation**. (A) Expression of all adrenergic receptor isoform encoding genes in indicated tissue macrophage populations. Expression values normalized using DESeq2 method were obtained from Immgen (n = 2–3 per sample). (B) Gene Set Enrichment Analysis of transcriptomic changes induced by fenoterol stimulation in BMDMs *in vitro* (data from GSE160640, normalized to unstimulated controls) to changes between indicated tissue macrophage populations and scWAT macrophages (data from GSE103847). ‘SAM fibers’ correspond to sympathetic nerve-associated macrophages in scWAT, while ‘SAM ganglia’ – to macrophages interacting with nerves in paravertebral sympathetic ganglia. Lower p value indicates higher similarities of changes between two datasets. (C) The expression of the top 80 most up-regulated genes by 1 h fenoterol treatment in BMDMs, measured in indicated tissue macrophage populations, expressed as fold change to scWAT macrophage expression value (data from Pirzgalska et al.).

To investigate the importance of adrenergic signaling in ATMs, we first examined the expression of the adrenergic receptor genes in different mouse tissue macrophage populations and WAT cell types. In accordance to other reports [13], *Adrb2* was the main adrenergic receptor gene isoform in tissue macrophages, with relatively high expression levels in the ATM population (Figure 1A). Publicly available single-cell RNAseq data [21] corroborated these findings, with approximately half of epididymal WAT (eWAT) myeloid cell population showing high *Adrb2* expression (Supplementary Figure S1). Conversely, the other adrenergic receptor gene isoforms with detectable levels in myeloid cells, namely *Adrb1*, *Adra2a* and *Adra2b*, were only expressed in less than 4% of cells within the eWAT myeloid population (Figure S1). Furthermore, in accordance with a previous study [13], *Adrb2* expression was enriched in CD11b-positive cells compared to other tissue-resident cells in both eWAT and subcutaneous WAT (scWAT), while *Adrb3* was mainly expressed in adipocytes (Supplementary Figure S2).

SNS-associated ATMs have been reported to express higher *Adrb2* levels compared to the overall ATM population [9]. We therefore hypothesized that due to their close proximity to sympathetic nerves and high *Adrb2* expression, SNS-associated ATMs would exhibit markers of B2AR activation. To test this, we compared the transcriptional changes induced by B2AR agonist fenoterol in bone marrow-derived macrophages (BMDMs) *in vitro* (GSE160640) to the transcriptome differences between macrophages and SNS-associated ATMs isolated from scWAT (GSE103847). Indeed, the transcriptional profile of SNS-associated ATMs, and not of other analyzed tissue macrophages, was highly similar to the profile induced by acute B2AR activation in BMDMs (Figure 1B–C). Overall, our findings suggested that the sympathetic nerves could signal to SNS-associated ATMs specifically via beta2-adrenergic receptor.

### 3.2 Macrophage-specific *Adrb2* deletion does not affect systemic energy balance on a chow or HFD

To disrupt the neurotransmission from the sympathetic nerves to ATMs, while leaving the SNS signaling to other adipose tissue cells intact, we deleted the *Adrb2* gene selectively in macrophages by crossing *Adrb2*^fl/fl^ and *Lyz2*^Cre/+^ mice. We have previously demonstrated that bone marrow-derived macrophages (BMDMs) from *Adrb2*^ΔLyz2^ mice are unresponsive to NE stimulation *in vitro* [22]. As we observed a potent downregulation of *Adrb2* mRNA in both scWAT and eWAT CD11b-positive cells isolated from *Adrb2*^ΔLyz2^ animals (Supplementary Figure S3), and as different tissue macrophages have a similar profile of adrenergic receptor encoding genes (Figure 1A), we reasoned that ATMs would be unresponsive to NE in *Adrb2*^ΔLyz2^ mice, similar to the previous findings on intestinal [6,7], splenic [23,24], brain [25], lung [26], and bone marrow [22] macrophages in this mouse model.

**Figure 2:**
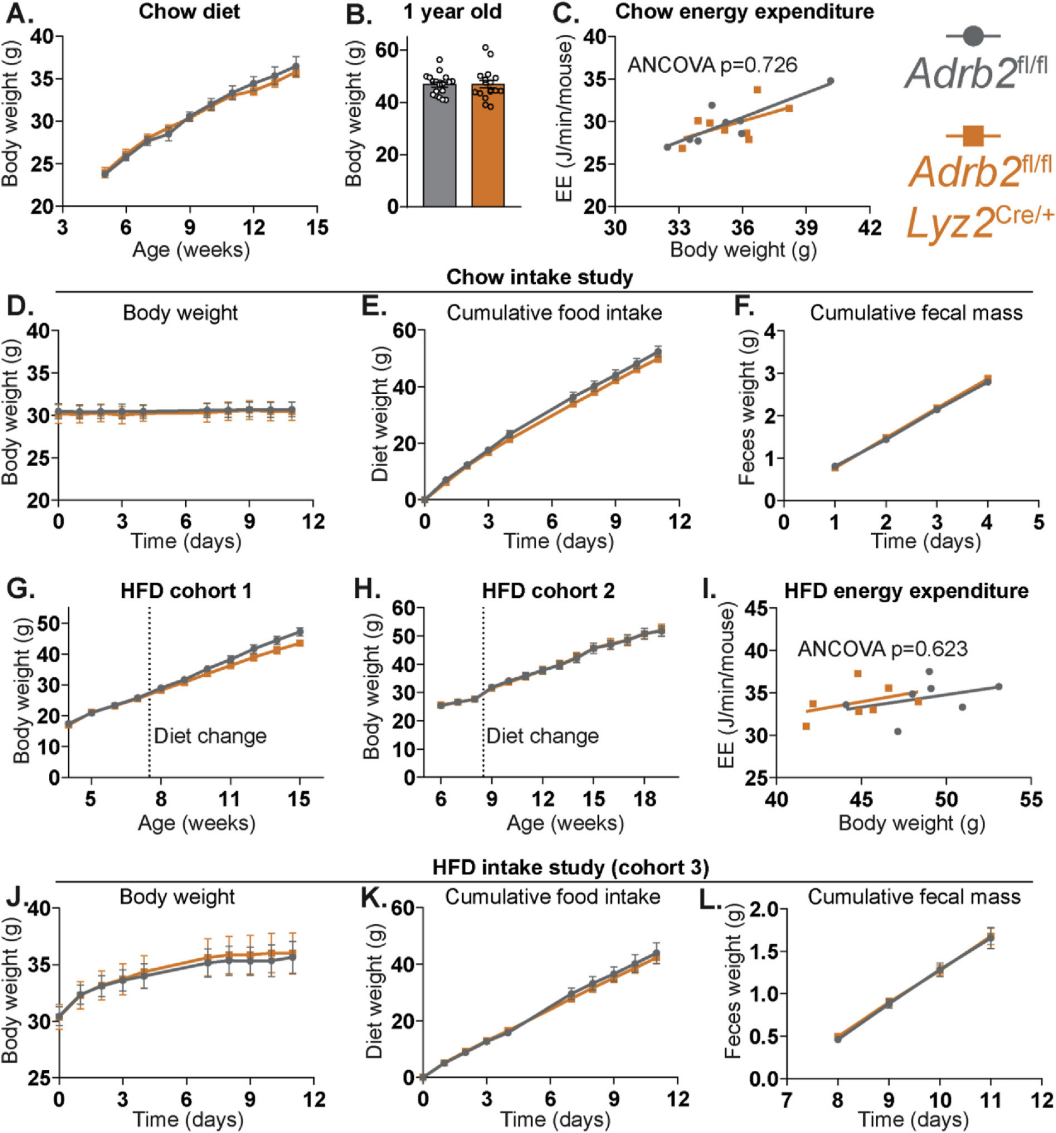
**Unaltered energy balance in macrophage-specific *Adrb2* knockout mice.** (A) Growth curves of *Adrb2*^fl/fl^ (n = 8) and *Adrb2*^fl/fl^*Lyz2*^Cre/+^ (n = 8) male mice on a chow-diet. (B) Weights of *Adrb2*^fl/fl^ (n = 19) and *Adrb2*^fl/fl^*Lyz2*^Cre/+^ (n = 17) chow-fed male mice at one year of age. (C) Energy expenditure of *Adrb2*^fl/fl^ (n = 8) and *Adrb2*^fl/fl^*Lyz2*^Cre/+^ (n = 8) chow-fed 4-month-old male mice. (D) Body weights and (E) cumulative chow intake of *Adrb2*^fl/fl^ (n = 8) and *Adrb2*^fl/fl^*Lyz2*^Cre/+^ (n = 8) mice, measured over 11-day period, and (F) cumulative fecal weight, measured over days 7–11 of the experiment. Growth curves of (G) the first cohort of *Adrb2*^fl/fl^ (n = 8) and *Adrb2*^fl/fl^*Lyz2*^Cre/+^ (n = 8) and the second cohort of *Adrb2*^fl/fl^ (n = 10) and *Adrb2*^fl/fl^*Lyz2*^Cre/+^ (n = 11) male mice on HFD. (I) Energy expenditure of the first cohort of *Adrb2*^fl/fl^ (n = 7) and *Adrb2*^fl/fl^*Lyz2*^Cre/+^ (n = 7) 4-month-old male mice after 9 weeks of HFD. (J) Body weights and (K) cumulative HFD intake of *Adrb2*^fl/fl^ (n = 8) and *Adrb2*^fl/fl^*Lyz2*^Cre/+^ (n = 8) mice, measured over the first 11 days after the change to HFD, and (L) cumulative fecal weight, measured over days 7–11 of the experiment. All graphs show means ± SEM.

We initially assessed energy balance in *Adrb2*^ΔLyz2^ mice, given that it is known to be impacted by both adipose tissue SNS tone and ATM phenotype [2,15]. *Adrb2*^ΔLyz2^ animals had comparable weight gain on a chow diet during early life and weighed similarly to controls at 1 year of age (Figure 2A–B). Furthermore, *Adrb2* expression in macrophages had no effect on energy expenditure, food intake, or fecal mass on chow diet (Figure 2C–F). While the first cohort of *Adrb2*^ΔLyz2^ mice tended to diverge in body weight from controls after switching them from chow to HFD (Figure 2G, differences not statistically significant), this finding could not be replicated in any of the subsequent cohorts (Figure 2H,J). Moreover, no differences in energy expenditure were observed between *Adrb2*^ΔLyz2^ and control mice during HFD (Figure 2I). Finally, both *Adrb2*^ΔLyz2^ and control groups demonstrated comparable weight gain, food intake, and fecal excretion during the initial two weeks of high-fat feeding (Figures 2J–L). Overall, we found that the absence of macrophage B2AR did not affect energy balance on a chow or HFD.

### 3.3 Macrophage-specific *Adrb2* deletion does not affect WAT function during fasting or refeeding

SNS outflow to adipose tissues is increased during fasting in order to promote adipocyte lipolysis and to provide energy in the form of fatty acids to other tissues [2]. Fasting is also known to stimulate macrophage recruitment to WAT and to promote ATM triglyceride accumulation [27,28]. As NE can both regulate macrophage chemotaxis [29] and increase macrophage triglyceride storage [22], we reasoned that the loss of *Adrb2* in macrophages could impair the recruitment of macrophages to WAT and reduce their intracellular lipid storage in response to fasting. This in turn would reduce ATM lipid buffering capacity, leading to increased WAT lipolysis, as shown previously in mice depleted of ATMs [27,30]. Finally, impaired WAT lipolysis during fasting can be associated with an impaired adipocyte lipid uptake, hyperlipidemia and hyperinsulinemia in the postprandial state [17].

**Figure 3:**
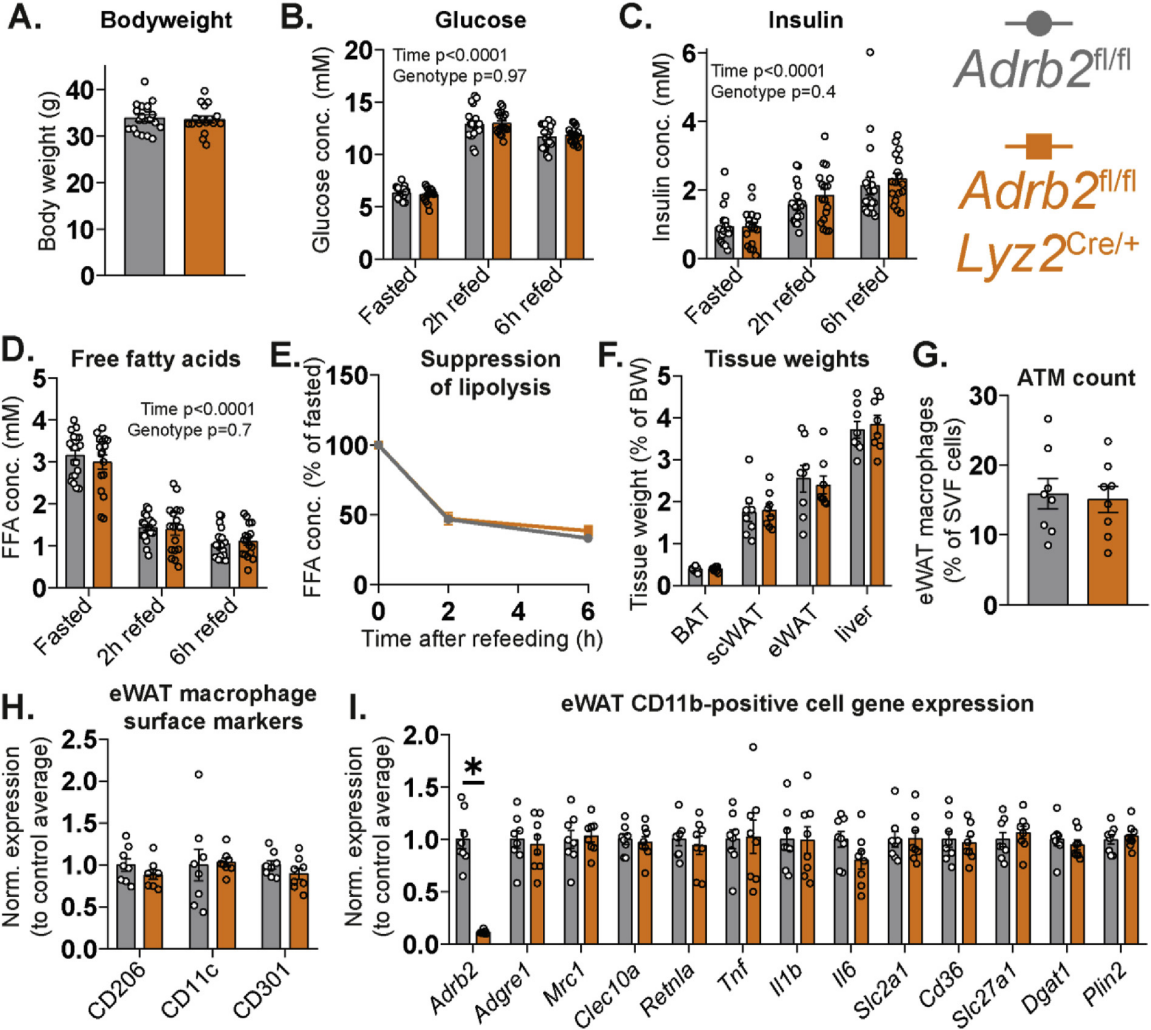
**Normal physiological response of macrophage-specific *Adrb2* knockout mice to fasting and refeeding.** (A) Weights of *Adrb2*^fl/fl^ (n = 20) and *Adrb2*^fl/fl^*Lyz2*^Cre/+^ (n = 17) chow-fed 3-month-old male mice after 16-hour fast. (B) Blood glucose, (C) serum insulin and (D) serum free fatty acids in the same mice after 16-hour fast and after refeeding for 2 and 6 h. (E) Suppression of lipolysis in response to refeeding, calculated by dividing the refed FFA values from the fasted value for each mouse. (F) Weights of indicated tissues isolated from *Adrb2*^fl/fl^ (n = 8) and *Adrb2*^fl/fl^*Lyz2*^Cre/+^ (n = 8) chow-fed 4-month-old male mice after 16-hour fast. (G) Flow cytometry quantification of eWAT macrophage number and (H) the expression of indicated surface proteins in the macrophage population, normalized to the average of *Adrb2*^fl/fl^ group. (I) qPCR expression analysis of indicated genes in the CD11b-positive eWAT cell fraction. All graphs show means ± SEM. In (B–D), p values of 2-way ANOVA are indicated on the graph. In (I), ∗ indicates p < 0.05 compared between groups using a two-tailed Student's *t*-test.

We subjected *Adrb2*^ΔLyz2^ and control mice to a 16-h fast, followed by a 6-h refeeding period with a chow diet. No differences in body weight, circulating glucose, insulin and free fatty acid levels were observed between *Adrb2*^ΔLyz2^ and control mice after an overnight fast (Figure 3A–D). Furthermore, both *Adrb2*^ΔLyz2^ and control groups demonstrated comparable increases in blood glucose and serum insulin in response to refeeding (Figure 3B–C). *Adrb2* expression in macrophages had no effect on the suppression of lipolysis following refeeding (Figure 3D–E). Tissue weights, ATM number and inflammatory phenotype were similar in *Adrb2*^ΔLyz2^ and control mice after 16-h fast (Figure 3F–H). Finally, no changes in the expression of macrophage polarization markers, lipid uptake and lipid storage marker genes were found in the CD11b-positive cells isolated from fasted *Adrb2*^ΔLyz2^ and control groups (Figure 3I). Altogether, the loss of B2AR in macrophages did not affect ATM recruitment or phenotype during fasting and did not modulate WAT function during fasting or refeeding.

### 3.4 Macrophage-specific *Adrb2* deletion does not affect thermoregulation, BAT or WAT function upon cold exposure

A drop in the environmental temperature increases the SNS outflow to both BAT and WAT in order to stimulate thermogenesis in BAT and promote lipolysis in WAT to supply fatty acids to BAT for oxidation [31]. With respect to the role of macrophage polarization in mediating the browning of WAT, there is conflicting evidence. On the one hand, cold exposure has been described to promote macrophage recruitment to WAT and induce an M2 polarization state in ATMs, which reputedly is required for scWAT browning and adaptive thermogenesis [32]. Conversely, cold exposure has been shown to increase the circulating levels of an anti-inflammatory cytokine interleukin-10 (IL-10), which reportedly inhibits WAT browning [33,34]. B2AR activation is known to potentiate M2 polarization in macrophages treated with an anti-inflammatory cytokine interleukin-4 (IL-4), as well as to promote macrophage IL-10 production and their acquisition of an inflammatory resolution phenotype in response to pro-inflammatory stimuli [35,36]. We confirmed these findings in BMDMs – co-stimulation with fenoterol potentiated the increase in M2 marker genes in IL-4-treated cells (Supplementary Figure S4). Similarly, fenoterol stimulation reduced the expression of a pro-inflammatory gene *Tnf*, encoding cytokine TNFα, while co-stimulation with fenoterol and lipopolysaccharide enhanced *Il10* and suppressed *Tnf* gene expression compared to LPS treatment alone (Supplementary Figure S5). We therefore hypothesized that increased ATM M2 polarization and the elevated circulating IL-10 levels observed in response to cold exposure could be attributed to macrophage B2AR signaling. Loss of B2AR in macrophages would therefore either potentiate (due to impaired IL-10 production) or inhibit (due to impaired M2 polarization) scWAT browning and thermogenesis during cold exposure.

**Figure 4:**
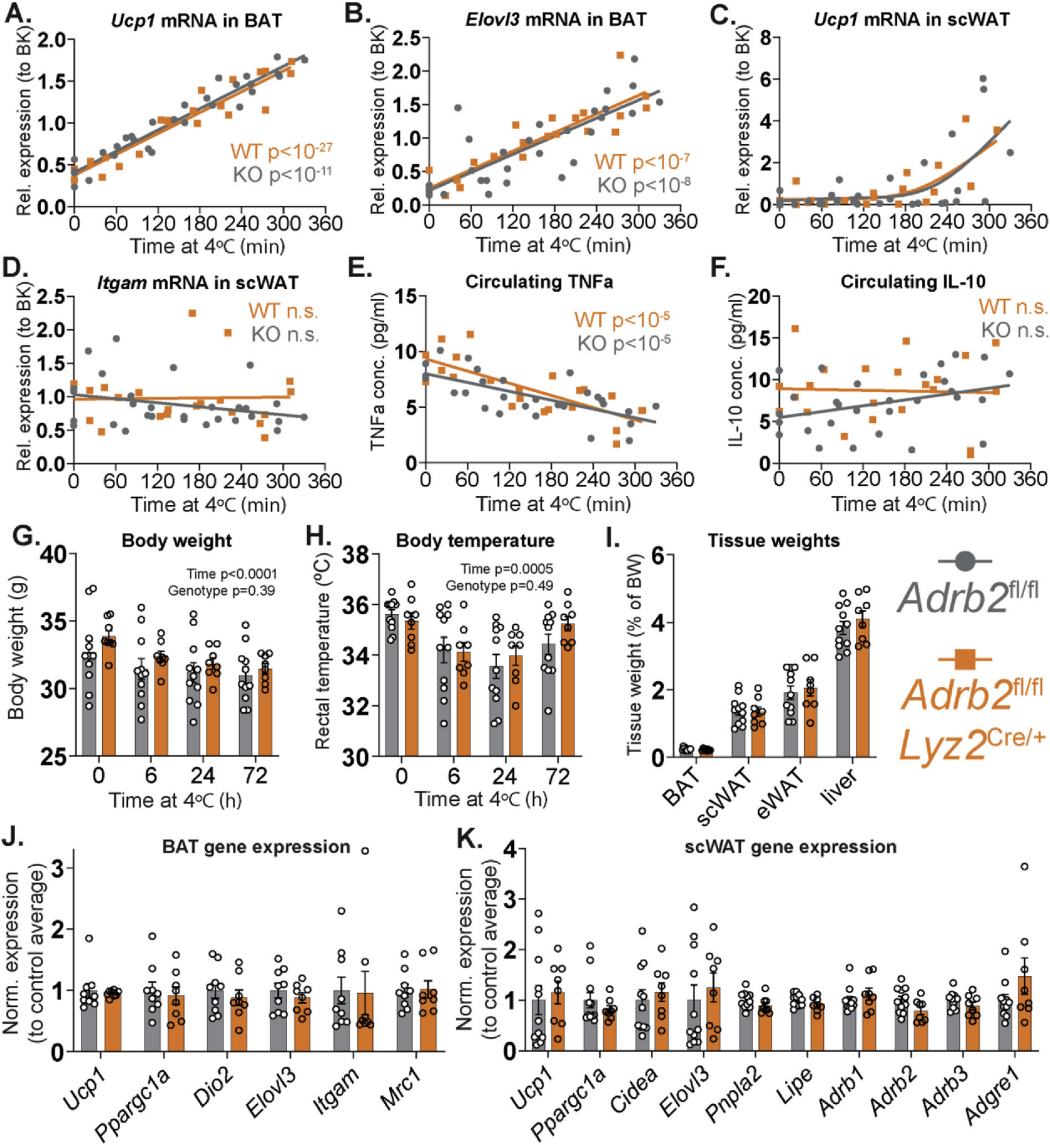
**Normal physiological response of macrophage-specific *Adrb2* knockout mice to cold exposure.** (A) *Ucp1* and (B) *Elovl3* mRNA in BAT, (C) *Ucp1* and (D) *Itgam* mRNA in scWAT and (E) serum TNFα and (F) IL-10 levels in *Adrb2*^fl/fl^ (n = 25 male, 4 female) and *Adrb2*^fl/fl^*Lyz2*^Cre/+^ (n = 18 male, 4 female) chow-fed 3-month-old mice, exposed to 4 °C for times varying between 0 and 5.5 h. (G) Body weights, (H) rectal temperature, (I) tissue weights and qPCR expression analysis of indicated genes in (J) BAT and (K) scWAT of *Adrb2*^fl/fl^ (n = 11) and *Adrb2*^fl/fl^*Lyz2*^Cre/+^ (n = 8) chow-fed 3-month-old male mice, exposed to 4 °C for 72 h. All graphs show means ± SEM. In (A-B, D-F), p values of linear regression and ANCOVA analyses are indicated on the graph. In (G–H), p values of two-way ANOVA are indicated on the graph.

As ATMs were reported to control sympathetic innervation in BAT [10], we initially checked whether SNS signaling in BAT in response to cold exposure was altered in *Adrb2*^ΔLyz2^ animals. To do so, we transferred the mice from the room temperature to 4 °C for times varying between 0 and 6 h and measured the levels of *Ucp1* mRNA in BAT, which is well-known to be upregulated in brown adipocytes in response to NE stimulation. The mRNA expression of *Ucp1* and another thermogenic adipocyte marker *Elovl3* showed a highly significant positive linear correlation with the duration of cold exposure (Figure 4A–B). Importantly, the induction of either *Ucp1* or *Elovl3* was comparable in both *Adrb2*^ΔLyz2^ and control groups (Figure 4A–B), indicating that B2AR deficiency in macrophages did not affect downstream markers of either the SNS outflow to BAT, or NE signaling in brown adipocytes.

Compared to BAT, the kinetics of *Ucp1* mRNA increase in scWAT were delayed, but were similar between *Adrb2*^ΔLyz2^ and controls (Figure 4C). We did not see changes in the scWAT expression of *Itgam* gene, encoding macrophage marker F4/80, during the initial 6 h of cold exposure in either study group (Figure 4D). In accordance to the paradigm that cold exposure promotes macrophage M2 polarization [32], circulating levels of TNFα were rapidly reduced in mice placed at 4 °C; however, this occurred independently of the B2AR signaling in macrophages (Figure 4E). Finally, no alterations in serum IL-10 levels were observed in either *Adrb2*^ΔLyz2^ or control groups during acute cold exposure (Figure 4F).

As the acute response to cold exposure was unaffected by macrophage-specific B2AR deletion, we decided to investigate whether *Adrb2*^ΔLyz2^ animals could maintain core body temperature and sustain scWAT browning for a longer duration in a cold environment. Both the *Adrb2*^ΔLyz2^ and control groups demonstrated comparable weight loss and core body temperatures during a three-day cold (4 °C) exposure protocol (Figure 4G–H). Following three days of cold exposure, no differences in adipose tissue or liver weights were observed between groups (Figure 4I). Both groups showed similar expression of thermogenic genes in BAT (Figure 4J). Finally, while the mRNA markers of beige adipocytes, lipolysis and ATMs in scWAT were variable, they were similar in *Adrb2*^ΔLyz2^ and control mice exposed to 4 °C for three days (Figure 4K). Overall, B2AR signaling in macrophages was not required for the adaptive physiological response to the cold exposure.

### 3.5 Macrophage-specific *Adrb2* deletion does not affect WAT inflammation and systemic insulin sensitivity on a chow or HFD

Thus far, we had observed normal BAT and WAT function in *Adrb2*^ΔLyz2^ mice during physiological challenges in lean states. As sympathetic nerve activity has been suggested to maintain ATMs in an anti-inflammatory state via macrophage B2AR signaling [13], and as sympathetic nerve-associated ATMs acquire greater pro-inflammatory phenotype than a general ATM population in response to high-fat feeding [9], we hypothesized that B2AR signaling in macrophages could be important to limit ATM M1 polarization and WAT inflammation in obesity. In accordance to such hypothesis, high-fat feeding progressively downregulated *Adrb2* expression in CD11b-positive cells (Supplementary Figure S6), similarly to an established obesity-related reduction in beta-adrenergic receptor levels on adipocytes [37,38]. Importantly, the decrease in *Adrb2* mRNA coincided with the loss of M2 macrophage marker gene expression in the CD11b-positive cell population (Supplementary Figure S6). Therefore, we reasoned that the deletion of B2AR in macrophages would accelerate the development of WAT inflammation, glucose intolerance, and insulin resistance during high-fat feeding.

**Figure 5:**
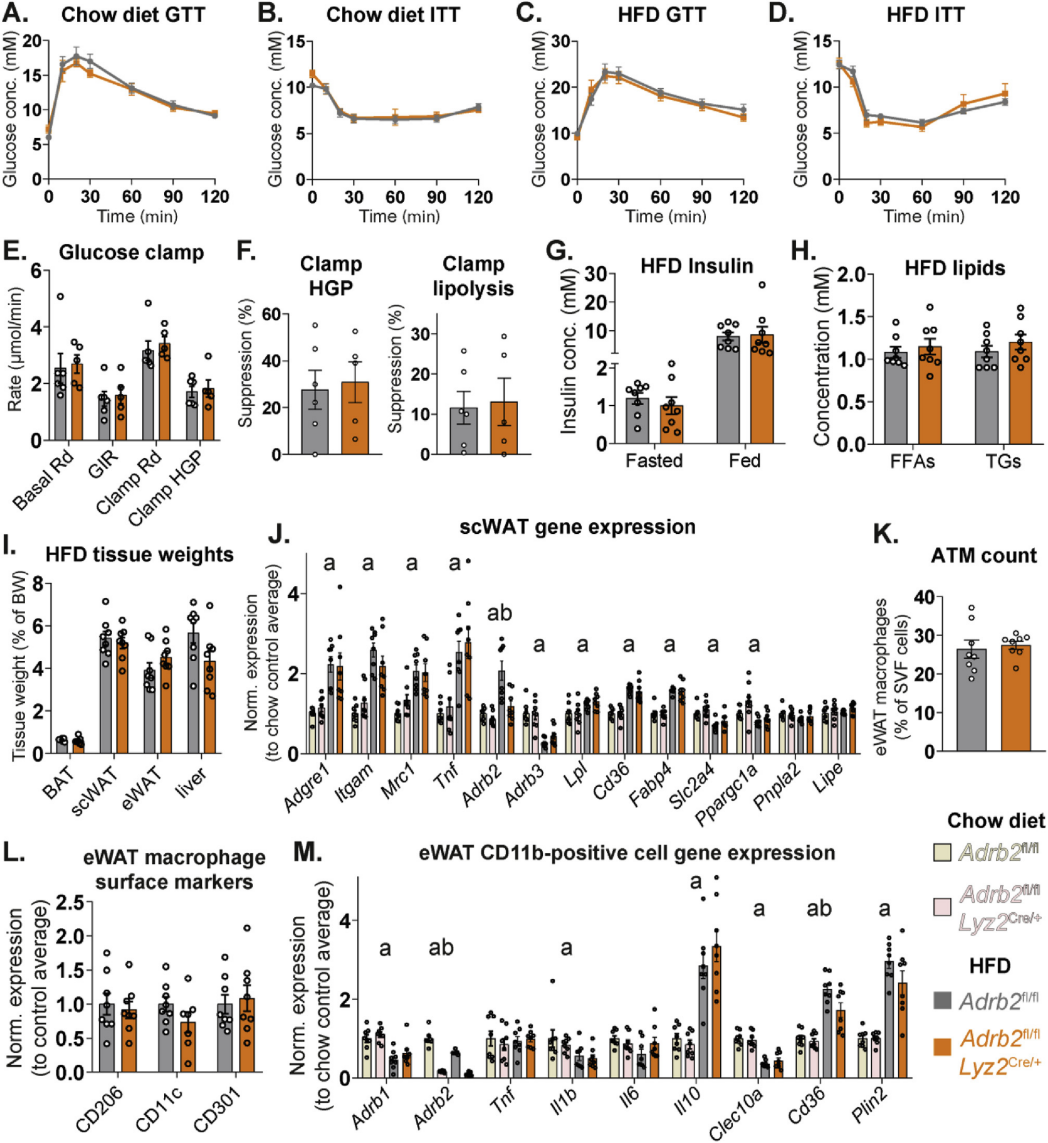
**Unaltered development of WAT inflammation and systemic insulin resistance in HFD-fed macrophage-specific *Adrb2* knockout mice.** Glucose excursion curves during intraperitoneal (A) glucose and (B) insulin tolerance tests in *Adrb2*^fl/fl^ (n = 8) and *Adrb2*^fl/fl^*Lyz2*^Cre/+^ (n = 8) chow-fed 5-month-old male mice. Glucose excursion curves during intraperitoneal (C) glucose and (D) insulin tolerance tests in *Adrb2*^fl/fl^ (n = 8) and *Adrb2*^fl/fl^*Lyz2*^Cre/+^ (n = 8) 5-month-old male mice, fed HFD for 10–11 weeks. (E) Glucose disposal rates (Rd) in basal and hyperinsulinemic (Clamp) states, glucose infusion rates and hepatic glucose production rate during hyperinsulinemic-euglycemic glucose clamp experiment in *Adrb2*^fl/fl^ (n = 6) and *Adrb2*^fl/fl^*Lyz2*^Cre/+^ (n = 5) 5-month-old male mice, fed HFD for 12 weeks. (F) Suppression of hepatic glucose production rate and lipolysis rate in the hyperinsulinemic state of glucose clamp experiment. (G) Serum insulin measured after 16-hour fast or in a random fed state, and (H) serum free fatty acid and triglyceride levels, measured in a random fed state in *Adrb2*^fl/fl^ (n = 8) and *Adrb2*^fl/fl^*Lyz2*^Cre/+^ (n = 8) 5-month-old male mice, fed HFD for 12 weeks. (I) Indicated tissue weights, (J) qPCR analysis of indicated gene expression in scWAT, (K) flow cytometry quantification of eWAT macrophage number and (L) the expression of indicated surface proteins in the macrophage population (normalized to the average of *Adrb2*^fl/fl^ group) (M) qPCR expression analysis of indicated genes in the CD11b-positive eWAT cell fraction from *Adrb2*^fl/fl^ (n = 8) and *Adrb2*^fl/fl^*Lyz2*^Cre/+^ (n = 8) 5-month-old male mice, fed HFD for 12 weeks. In (J, M), samples were analyzed together with corresponding *Adrb2*^fl/fl^ (n = 8) and *Adrb2*^fl/fl^*Lyz2*^Cre/+^ (n = 8) age-matched male chow-fed control samples, and data was normalized to *Adrb2*^fl/fl^ chow-fed control group average. All graphs show means ± SEM. In (J, M), a indicates p < 0.05 for diet effect factor and b indicates p < 0.05 for genotype effect factor in a two-way ANOVA.

We tested our hypothesis in *Adrb2*^ΔLyz2^ and control mice fed HFD for three months. No differences in either glucose clearance during glucose tolerance tests, or the glucose lowering effect of insulin were observed between genotypes in HFD-fed or age-matched chow-fed control groups (Figure 5A–D). Both *Adrb2*^ΔLyz2^ and control HFD-fed mice displayed comparable rates of endogenous glucose production and clearance during hyperinsulinemic-euglycemic clamp experiment (Figure 5E). Furthermore, the suppression of hepatic glucose production and of WAT lipolysis in response to hyperinsulinemia was similar between *Adrb2*^ΔLyz2^ and control mice, indicating a comparable degree of adipose tissue insulin resistance between the two groups on HFD (Figure 5F). B2AR signaling in macrophages did not affect the serum insulin levels in either overnight fasted or fed states, nor did it alter circulating free fatty acid and triglyceride levels in the fed state (Figure 5G–H). No differences in adipose tissue or liver weights were observed between *Adrb2*^ΔLyz2^ and control groups after three months of high-fat feeding (Figure 5I). HFD increased the expression of ATM and inflammatory marker genes in scWAT to a similar degree in both groups (Figure 5J). HFD-induced changes in metabolic gene expression in scWAT were comparable between genotypes (Figure 5J). Interestingly, the upregulation of *Adrb2* expression in scWAT in response to high-fat feeding did not occur in *Adrb2*^ΔLyz2^ mice (Figure 5J), suggesting that this increase during HFD could be attributed to the increased tissue number of ATMs, which express high levels of *Adrb2*. No differences in the ATM number or their surface polarization markers in eWAT were observed between *Adrb2*^ΔLyz2^ and control groups on HFD (Figures 5K–L). While a small decrease in the expression of fatty acid transporter *Cd36* gene was observed in the CD11b-positive cells isolated from *Adrb2*^ΔLyz2^ mice compared to controls, the expression of other measured genes were similar between the CD11b-positive cells from both genotypes on either chow or HFD (Figure 5M). Lastly, HFD feeding resulted in a higher expression of macrophage marker and *Tnf* genes in the adipocyte fraction of eWAT, indicating an increased presence of floating lipid-laden ATMs in HFD-fed mice (Supplementary Figure S7). While the *Adrb2*^ΔLyz2^ group tended to have reduced expression of macrophage marker genes in the adipocyte fraction compared to controls, the differences were not statistically significant (Supplementary Figure S7). Altogether, our results indicate that B2AR signaling in macrophages did not affect the development of WAT inflammation and insulin resistance during obesity.

### 3.6 Macrophage-specific *Adrb2* deletion does not affect the development of atherosclerosis on the *Ldlr*^*−/−*^ genetic background

Finally, compared to room temperature, housing mice at thermoneutrality has been reported to increase perivascular WAT inflammation, leading to the accelerated development of atherosclerosis on an atherogenic background, but with no differences in the systemic insulin sensitivity [39,40]. Reducing housing temperature increases systemic SNS tone in mice, and some phenotypic differences between mice housed at thermoneutrality and room temperature can be attributed to B2AR signaling [[41], [42], [43]]. We therefore hypothesized that the protective effect of room temperature housing on the atherosclerosis development in mice could be mediated by the SNS signaling to macrophages via B2ARs. Consequently, animals lacking B2ARs in macrophages would exhibit accelerated development of atherosclerosis compared to controls at room temperature.

**Figure 6:**
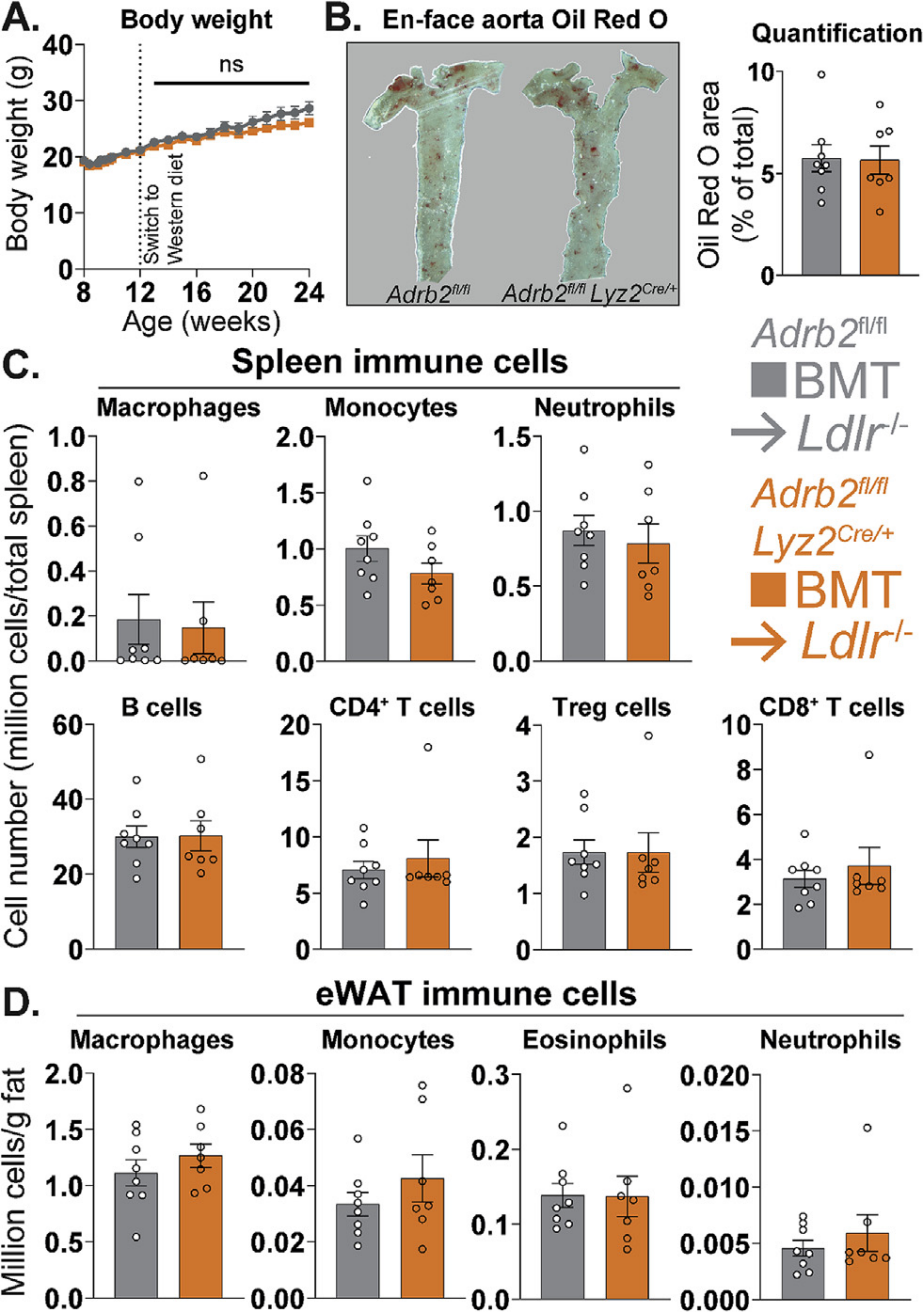
**Unaltered development of atherosclerosis and metaflammation in LDL receptor-null mice carrying macrophage-specific *Adrb2* knockout bone marrow.** (A) Body weights following the bone marrow transplantation and (B) representative images and Oil Red O area quantification of en face aortic plaques in *Ldlr*^−/-^ host mice carrying *Adrb2*^fl/fl^ (n = 8) and *Adrb2*^fl/fl^*Lyz2*^Cre/+^ (n = 7) bone marrow, placed on WD for 12 weeks. (C) Flow cytometry counts of indicated cell types in spleen, expressed as cell number per total spleen, or (D) in eWAT, expressed as millions of cells per g of fat, in the same experiment. Flow cytometry gating is described in the Methods section. All graphs show means ± SEM. No results are significantly different between genotypes using either a (A) repeated measures two-way ANOVA or (B–D) two-tailed Student's *t*-test.

To test our hypothesis, we transplanted *Adrb2*^ΔLyz2^ and control bone marrow to irradiated *Ldlr*^−/-^ host mice. We chose *Ldlr*^−/-^ female host mice for this experiment, as our previous studies demonstrated a significant development of atherosclerotic plaques in female mice of this genetic background fed atherogenic diet for 12 weeks [44,45]. Furthermore, we chose a Western diet that contains substantially more cholesterol than HFD and is routinely used in our studies to promote atherosclerotic plaque development [44,45]. There were no differences in body weights between the *Ldlr*^*−/−*^ mice carrying *Adrb2*^ΔLyz2^ and control bone marrow throughout the study (Figure 6A). At the end of experiment, both groups showed comparable sizes of aortic atherosclerotic plaques (Figure 6B). Furthermore, the splenic immune cell composition, which is a well-described factor affecting the development of atherosclerosis in mice [46], was similar in both genotypes (Figure 6C). Lastly, and in line with our previous findings from the HFD study, the myeloid cell composition of eWAT was unchanged in *Ldlr*^*−/−*^ mice carrying *Adrb2*^ΔLyz2^ bone marrow compared to controls (Figure 6D). Overall, our data demonstrate that B2AR signaling in macrophages did not modulate the progression of atherosclerosis or metaflammation in mice.

## 4 Discussion

In this study, we have investigated the physiological importance of sympathetic nerve-mediated NE-dependent signaling to ATMs for adipose tissue function and whole organism metabolic health. Having established that B2AR is the main adrenergic receptor in ATMs and that sympathetic nerve-interacting ATMs exhibit markers of B2AR activation, we generated mice with macrophage-specific B2AR deletion. However, these mice had no observable ATM phenotype, resulting in normal WAT function and systemic metabolism during multiple experimental paradigms including feeding, fasting, cold exposure, or obesity. Overall, we found that NE signaling to macrophages does not impact adipose tissue inflammation or function in either lean or obese states.

Our findings somewhat contradict the current view, which outlines the presence of a bidirectional communication between the sympathetic nerves and macrophages within adipose tissues [2,15]. Abundant evidence indicates that ATMs can affect SNS outflow to adipose tissues by either locally modulating nerve development [10,12], degrading NE [9,11] or by producing some yet unidentified sympathomimetic signaling factors [47]. However, the view that the SNS can directly modulate ATM phenotype is based on either pharmacologically stimulating SNS outflow to WAT [14] or surgically denervating mouse fat pads and observing either decreased or increased ATM pro-inflammatory activation [13], respectively. While these studies were informative, the main issue skewing their interpretation was that such experimental methods also modulated the SNS outflow to adipocytes. Therefore, the observed effects on ATM phenotypes could be attributed to either the changes in the direct SNS signaling to macrophages, or to the modulation of adipocyte phenotype occurring due to altered SNS activity. Given the findings presented in this paper, we propose that the sympathetic nerve activity indirectly maintains ATMs in an anti-inflammatory state by modulating the phenotype of adipocytes. In accordance to our conclusion, numerous adipocyte-targeted genetic mouse models with altered adipocyte survival [48], lipogenesis [47], lipolysis [49], lipid storage [50], mitochondrial function [51,52] and thermogenesis [[53], [54], [55]] show changes in adipose tissue inflammation and ATM phenotypes.

Alternatively, it is plausible that the sympathetic neurons do signal to the ATMs in a NE-independent manner. Intestinal neurons secrete CSF1 to promote the survival of MMs [5]. Furthermore, metabolites released from the neurons have been shown to affect the phenotype of neighboring glial cells [56]. Future investigations aimed to further define the neuroimmune interactions in adipose tissues are thus warranted.

It is important to emphasize that our findings do not contradict with the potential of B2AR agonists to reduce monocyte activation and pro-inflammatory responses in the kidneys and heart during obesity and type-2 diabetes, as previously reported [57]. While our data show that endogenous macrophage B2AR signaling does not regulate local or systemic inflammation in lean and obese states, it might still be stimulated pharmacologically above its physiological threshold level to reduce inflammation and alleviate the severity of metabolic disease. Given that B2AR agonists have also been shown to improve systemic insulin sensitivity in obese animals through their action on skeletal muscle cells [58], in future it would be of interest to compare the efficacy of B2AR agonists in *Adrb2*^ΔLyz2^ and control mice, in order to see what beneficial anti-inflammatory effects can be attributed to B2AR signaling specifically in macrophages, as compared to other cell types expressing B2ARs.

While we have observed a highly significant transcriptional signature of B2AR activation in macrophages interacting with sympathetic nerves in scWAT, it is plausible that such an observation could be an artifact arising from the isolation of these cells. The isolation process involves digesting scWAT sympathetic nerve fibers at 37 °C for 45 min, before sorting the macrophages from the resulting single-cell suspension [9]. Therefore, digestion might result in a breakdown of the sympathetic nerve fibers, release of NE into the medium and the activation of B2AR in macrophages present in the suspension. A ‘RiboTag’ genetic mouse model has been utilized in the past to overcome the isolation artifacts and analyze the cell-specific translatomes in a whole tissue [6]. However, at present there are no available tools to compare the transcriptional profiles of two macrophage populations in the same fat pad – namely ATMs that interact with the sympathetic neurons and those that do not – without tissue digestion and cell sorting.

Another limitation of our study was that we only investigated the phenotype of macrophages isolated from eWAT depots of *Adrb2*^ΔLyz2^ and control mice. As there are intrinsic biological differences between scWAT and eWAT depots [59], we could have overlooked some phenotypic changes that might have specifically occurred in macrophages residing in scWAT, but not in eWAT. However, both scWAT and eWAT contain an abundance of sympathetic nerve fibers [60], and sympathetic nerve-associated macrophages have been observed both in scWAT [9] and eWAT [11]. Furthermore, we found similar levels of *Adrb2* expression in CD11b-positive cells isolated from either scWAT or eWAT (Supplementary Figure S3). Overall, we believe that eWAT-resident macrophage is a good representative cell type to study the importance of macrophage adrenergic receptor signaling in adipose tissue metabolism.

While we found no changes in the expression of inflammatory genes, we observed a down-regulation of *Cd36* gene in the CD11b-positive cells isolated from HFD-fed *Adrb2*^ΔLyz2^ mice compared to controls. *Cd36* encodes a cell surface lipid transporter, which is enriched on lipid-laden ATMs isolated from obese humans and has been reported to promote adipose tissue inflammation in mice [61,62]. Furthermore, the expression of macrophage marker genes in the adipocyte fraction of eWAT, corresponding to lipid laden macrophages [63], tended to be reduced in *Adrb2*^ΔLyz2^ group compared to controls on HFD. As we previously reported that B2AR signaling promotes triglyceride storage in macrophages [22], it is plausible that B2AR signaling is in part responsible for obesity-related lipid accumulation in ATMs. However, fasting has also been reported to promote lipid storage in ATMs [27,28], and we found no differences in *Cd36* or other triglyceride storage-related gene expression in CD11b-positive cells isolated from fasted *Adrb2*^ΔLyz2^ mice compared to controls. Furthermore, lipid accumulation in ATMs has recently been shown to be uncoupled from the development of adipose tissue inflammation and insulin resistance [64]. Overall, we believe that further investigation of the role of B2AR signaling on the formation of lipid-laden ATMs would have little relevance to the function of adipose tissues and systemic metabolism, and is therefore not warranted based on our results and current literature.

Finally, as we have found that loss of B2AR in macrophages did not affect the ATM phenotype or WAT function in lean or obese states, it is interesting to speculate why ATMs express relatively high levels of *Adrb2* gene. So far, all the phenotypes that have been previously observed in *Adrb2*^ΔLyz2^ animals are related to an injury or infection. Specifically, *Adrb2*^ΔLyz2^ mice were shown to be protected against the lung inflammation and thrombosis occurring in response to particulate matter inhalation [26], but they were more susceptible to the heart [23,24] or brain injury [25] during myocardial infarction and stroke, respectively. Furthermore, loss of *Adrb2* in macrophages resulted in an inability to resolve inflammation following the gut bacterial infection [7] or systemic endotoxemia [36]. Therefore, perhaps B2AR signaling in ATMs is required for the repair of adipose tissue following a physical injury. Alternatively, macrophage B2ARs could also be important to regulate adipose tissue function during systemic bacterial or viral infections. While these are interesting and important biological questions that could be studied in future, their relevance to the obesity epidemic and the metabolic disease appears limited.

## Authors' contributions

K. Petkevicius, S. Virtue and A. Vidal-Puig conceptualized the study. K. Petkevicius, G. Bidault and S. Virtue performed research. S. Newland, G. Bidault and Z. Mallat performed atherosclerosis experiment and analysis. A. Dugourd and J. Saez-Rodriguez performed transcriptomic analysis. M. Dale provided technical assistance in experiments. S. Virtue and A. Vidal-Puig supervised the study. K. Petkevicius wrote the paper.
