# Supplementary material for: Macrophage beta2-adrenergic receptor is dispensable for the adipose tissue inflammation and function
Source: Mol Metab. 2021 Mar 25;48:101220. doi: 10.1016/j.molmet.2021.101220 (PMC8086137; doi:10.1016/j.molmet.2021.101220)
Supplement: Supplementary file 1 — Multimedia component 1 [file mmc1.docx]

**Supplementary Figure S1**
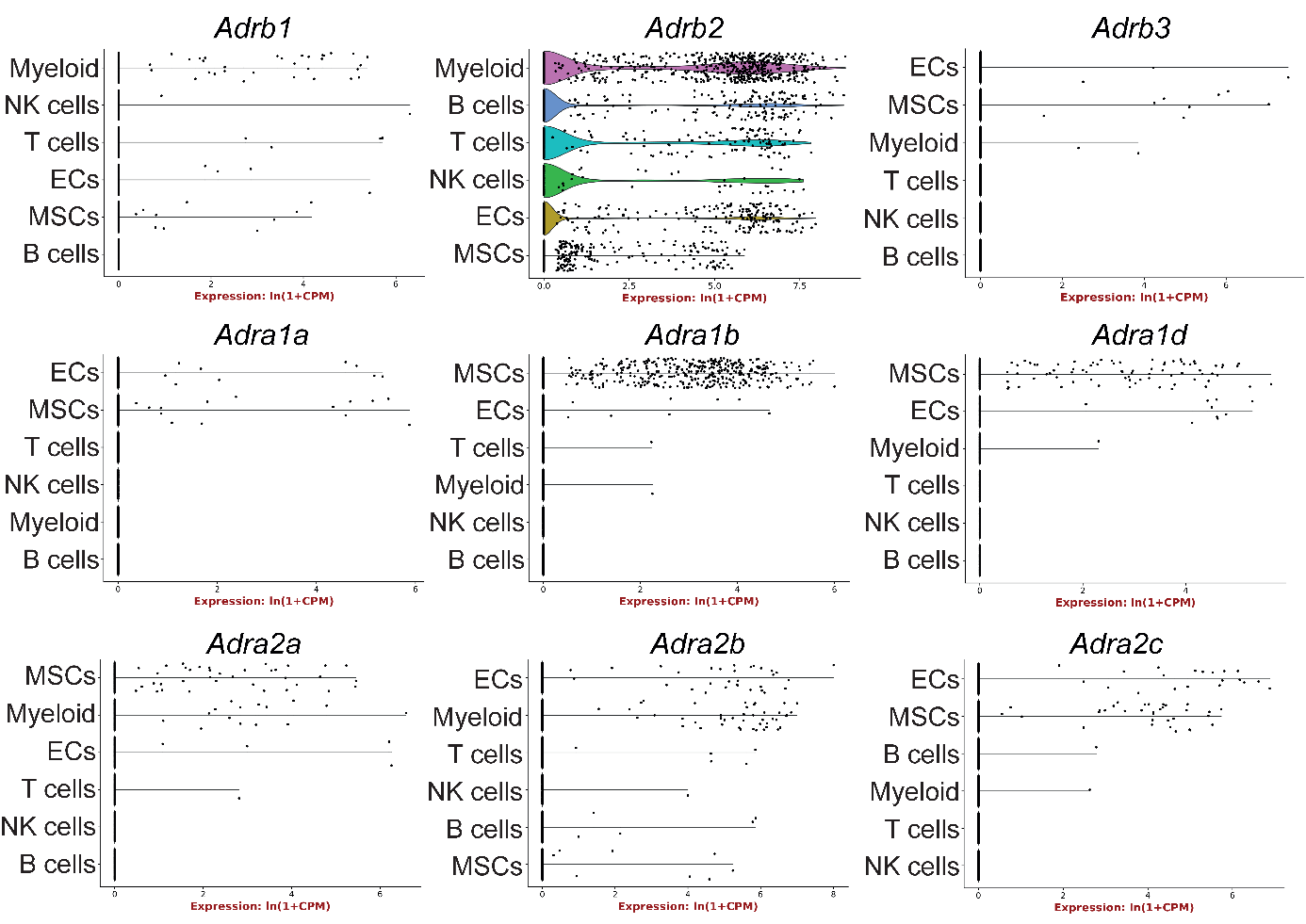


**Supplementary figure S1.** Violin plots of single-cell expression of adrenergic receptor encoding genes in the indicated mouse eWAT cell populations. Expression values for genes indicated above graphs are in ln(1+counts per million). Graphs were obtained from <https://tabula-muris.ds.czbiohub.org/>. NK – natural killer, EC – endothelial cells, MSCs- mesenchymal stem cells.

**Supplementary Figure S2**


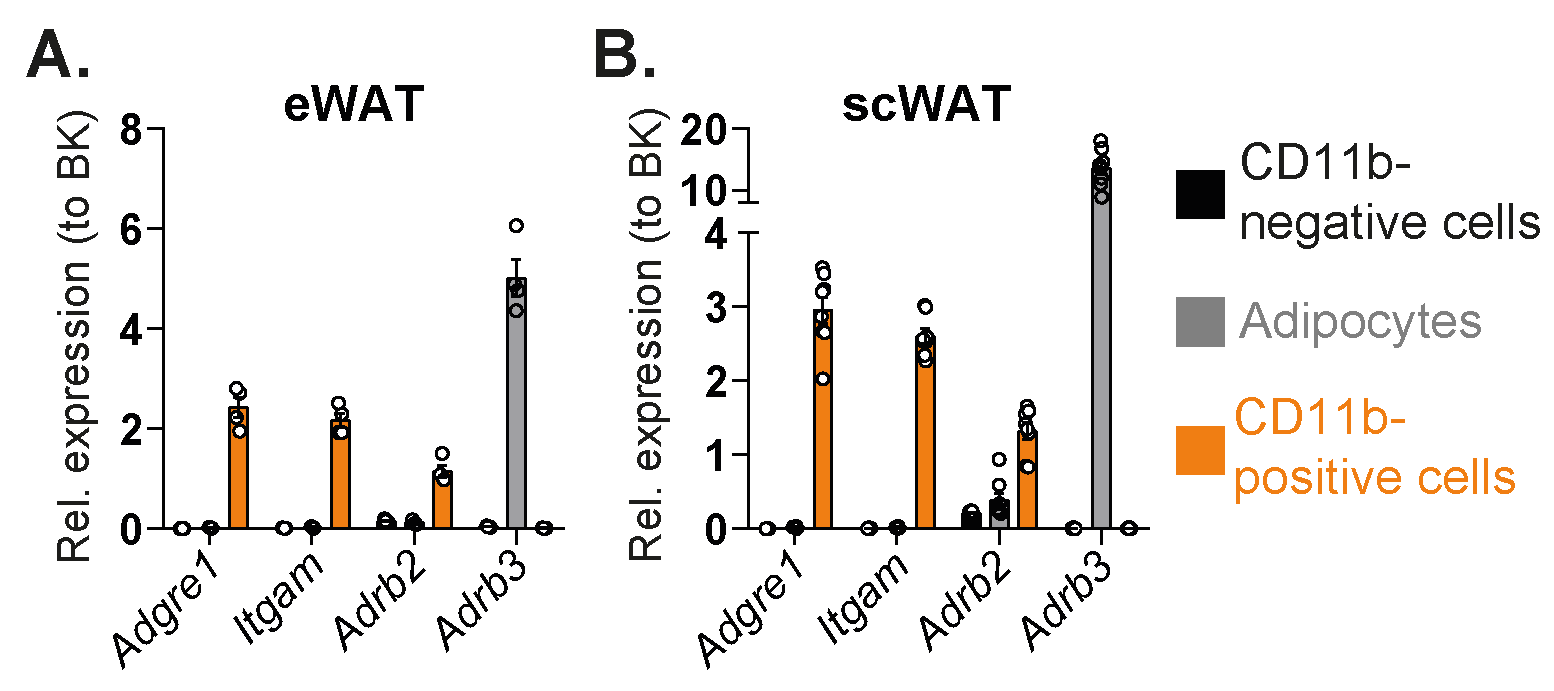


**Supplementary figure S2.** qPCR expression analysis of *Adgre1* and *Itgam* (encoding macrophage markers F4/80 and CD11b, respectively), and *Adrb2* and *Adrb3* genes in floating adipose tissue fraction (adipocytes), CD11b-positive or CD11b-negative cells isolated from eWAT (n=4) or scWAT (n=8) of chow-fed WT 3-month old male mice. All graphs show means ± SEM.

**Supplementary Figure S3**


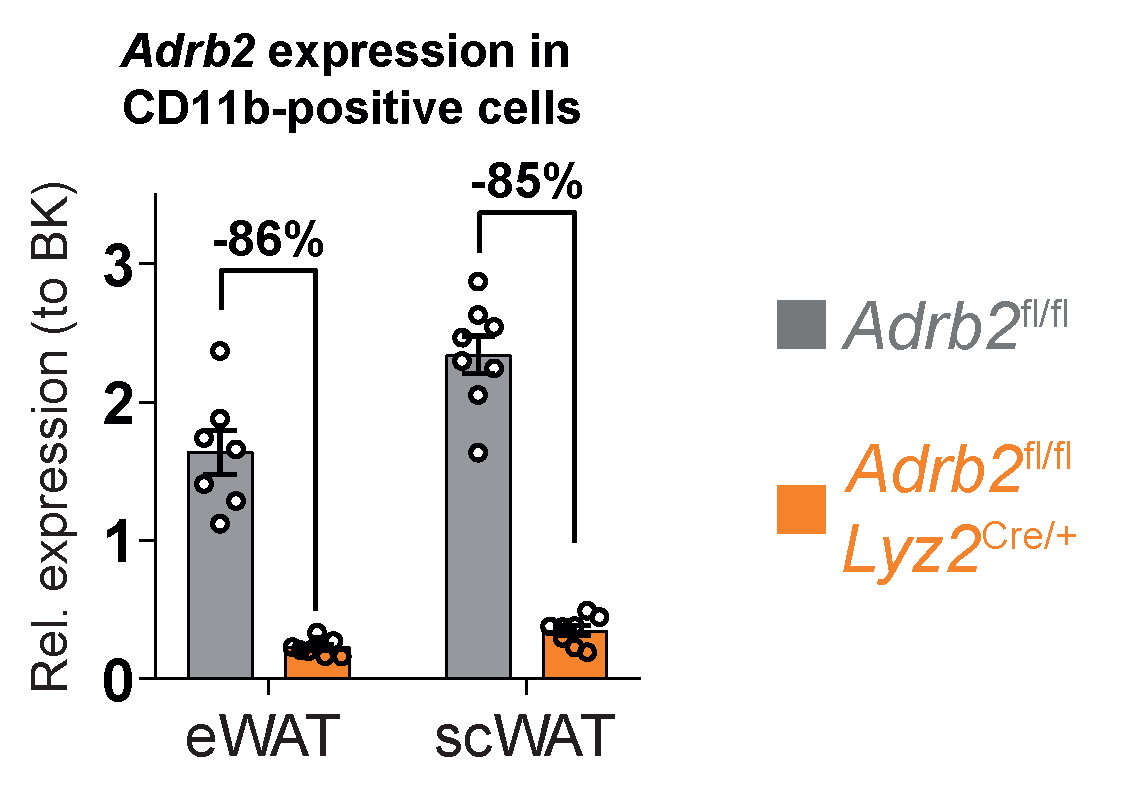


**Supplementary figure S3.** qPCR expression analysis of *Adrb2* in CD11b-positive cells isolated from eWAT and scWAT of *Adrb2*^fl/fl^ (n=7-8) and *Adrb2*^fl/fl^ *Lyz2*^Cre/+^ (n=8) chow-fed 3-month old male mice. Graph shows means ± SEM. Percentage values indicate the decrease in *Adrb2* expression in *Adrb2*^fl/fl^ *Lyz2*^Cre/+^ group relative to the respective *Adrb2*^fl/fl^ control group average.

**Supplementary Figure S4**


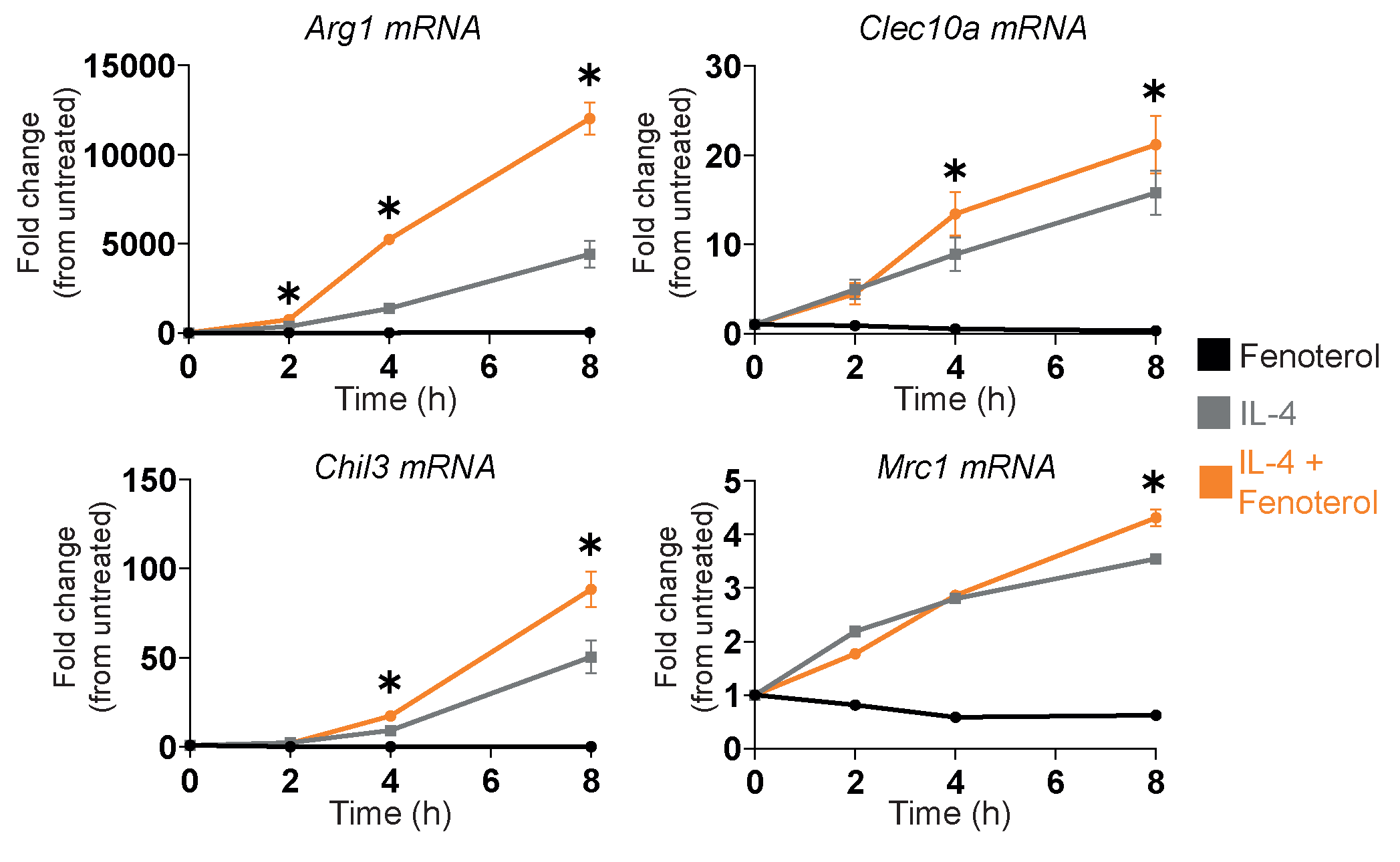


**Supplementary figure S4. B2AR activation potentiates the expression of M2 marker genes in response to IL-4 in BMDMs.** BMDMs were stimulated with either 1 µM fenoterol alone, with 10 ng/mL IL-4 or with both compounds simultaneously for 0, 2, 4 and 8 h, and the expression of indicated M2 marker genes was measured by qPCR. Gene expression values were normalized to the average of unstimulated control group. N=4 mice. All graphs show means ± SEM. * indicates p < 0.05 compared between IL-4 alone and IL-4 + fenoterol groups at each timepoint using repeated measures 2-way ANOVA with Tukey’s post hoc test.

**Supplementary Figure S5**


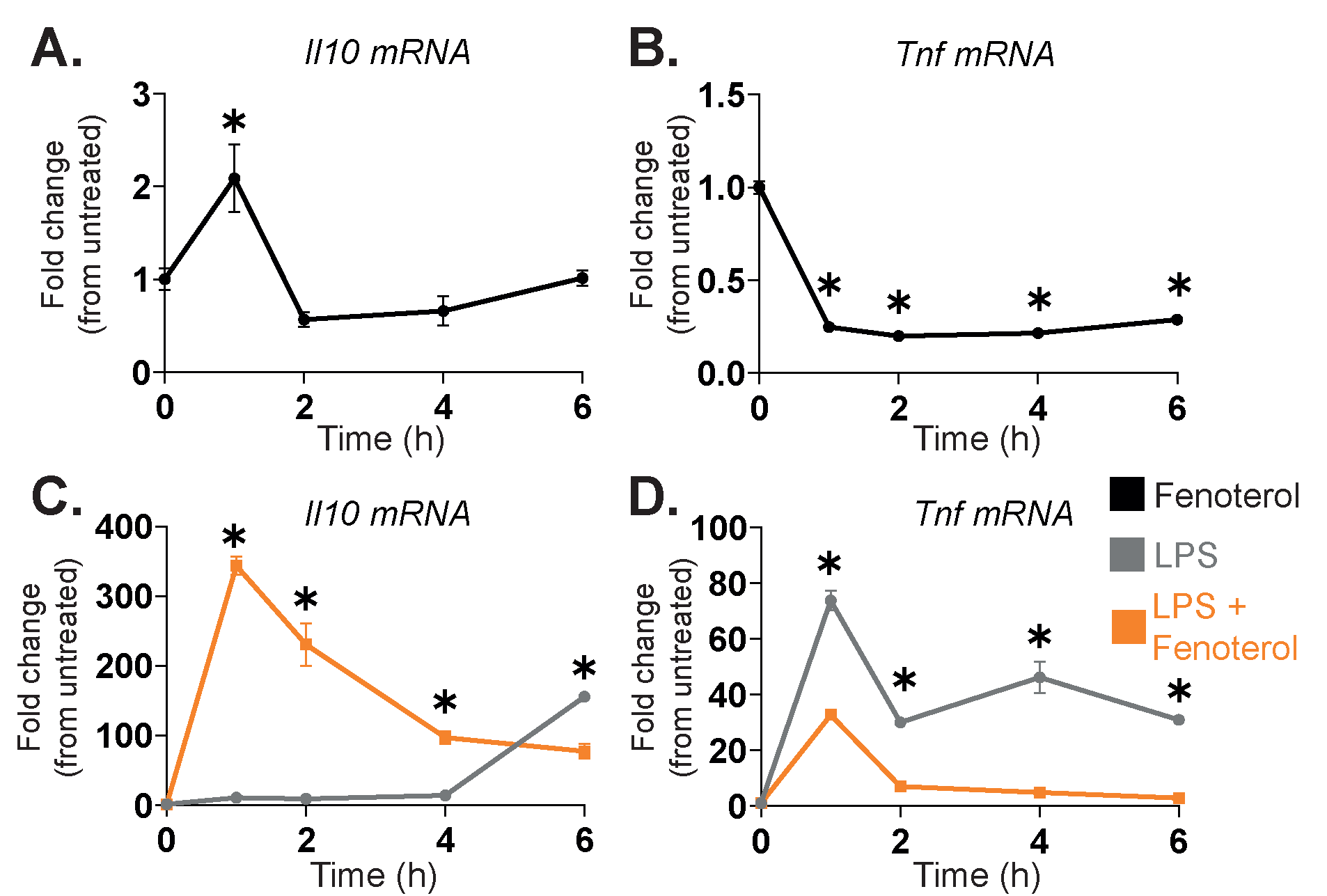


**Supplementary figure S5. B2AR activation induces a pro-resolution phenotype in BMDMs stimulated with LPS.** BMDMs were stimulated with either (A-B) 1 µM fenoterol alone, or (C-D) with 100 ng/mL LPS or with both compounds simultaneously for 0, 1, 2, 4, and 6 h, and the expression of *Il10* and *Tnf* genes was measured by qPCR. n=4 mice. Gene expression values were normalized to the average of unstimulated control group. All graphs show means ± SEM. In (A-B), * indicates p < 0.05 compared between each timepoint and control 0-hour timepoint using 1-way ANOVA with Dunnett’s post hoc test. In (C-D) * indicates p < 0.05 compared between LPS alone and LPS + fenoterol groups at each timepoint using repeated measures two-way ANOVA with Tukey’s post hoc test.

**Supplementary Figure S6**


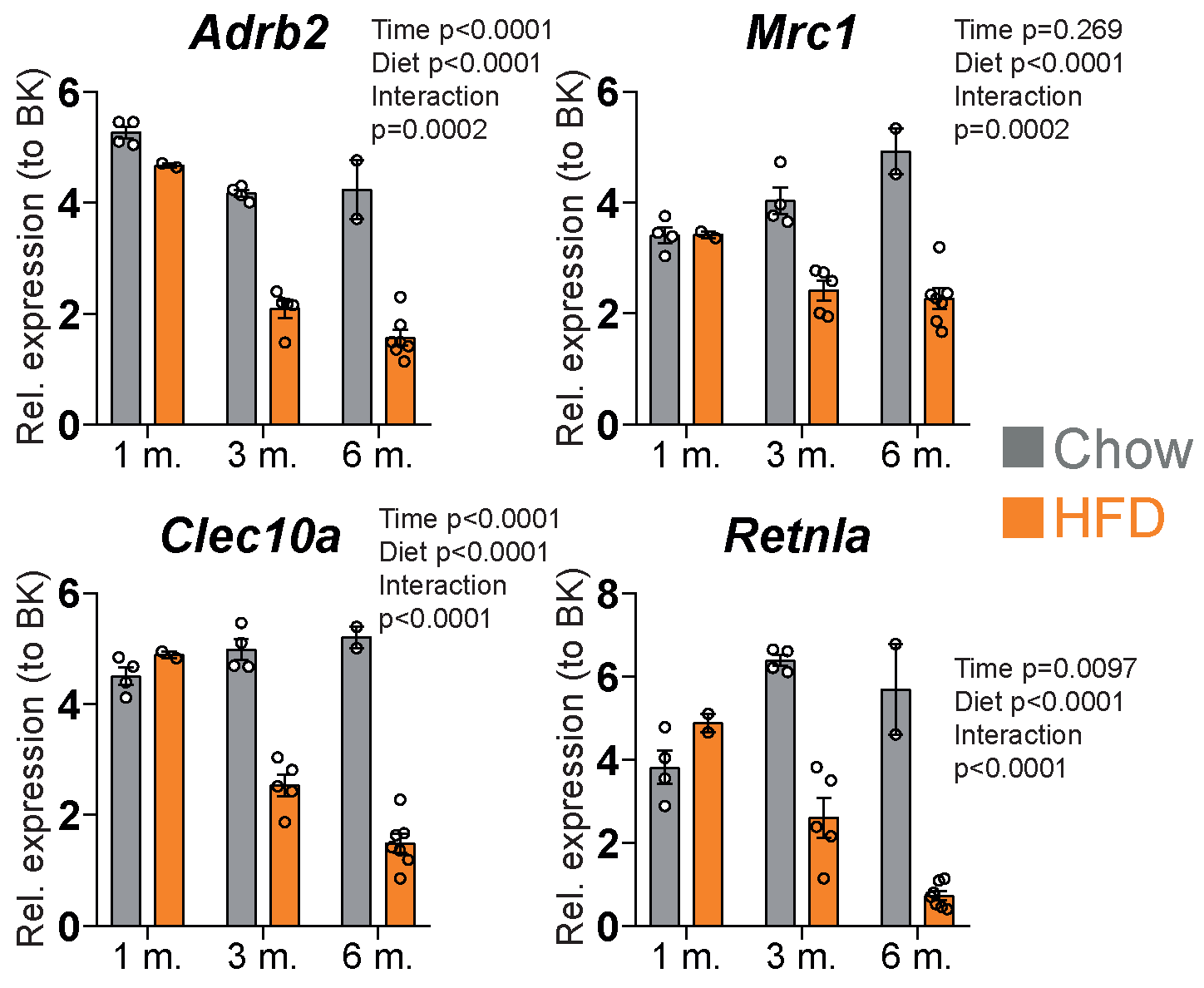


**Supplementary figure S6. HFD feeding downregulates *Adrb2* and M2 macrophage marker gene expression in ATMs.** qPCR analysis of *Adrb2* and indicated M2 macrophage marker gene expression in eWAT CD11b-positive cells isolated from male WT mice fed HFD for 1 (n=2), 3 (n=5) or 6 (n=7) months from 8 weeks of age, or their chow-fed age-matched controls (n=4 for 1 month, n=4 for 3 months and n=2 for 6 months). All graphs show means ± SEM. P values of two-way ANOVA are presented next to the graphs.

**Supplementary Figure S7**


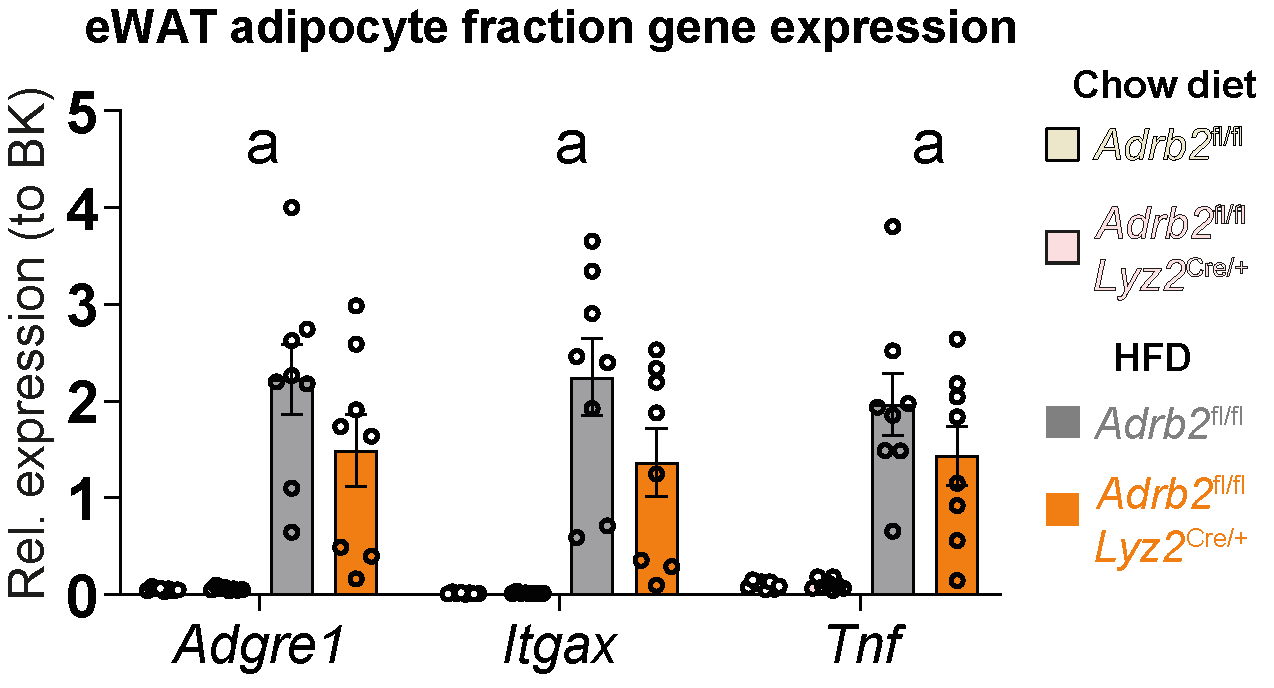


**Supplementary figure S7. Macrophage-specific *Adrb2* deletion does not alter the expression of macrophage marker and *Tnf* gene expression in the eWAT adipocyte fraction.** qPCR analysis of indicated genes in the eWAT adipocyte fraction from *Adrb2*^fl/fl^ (n=8) and *Adrb2*^fl/fl^ *Lyz2*^Cre/+^ (n=8) 5-month-old male mice, fed HFD for 12 weeks, and chow-fed age-matched control *Adrb2*^fl/fl^ (n=8) and *Adrb2*^fl/fl^ *Lyz2*^Cre/+^ (n=8) mice. “a” indicates p < 0.05 for diet effect factor and “b” indicates p < 0.05 for genotype effect factor in a two-way ANOVA.

**Supplementary TableS1**

| **Gene** | **Forward primer (5’-3’)** | **Reverse primer (5’-3’)** | **Probe (5’-3’)** |
| --- | --- | --- | --- |
| *18s* | CGGCTACCACATCCAAGGAA | GCTGGAATTACCGCGGCT | GAGGGCAAGTCTGGTGCCAG |
| *36b4* | AGATGCAGCAGATCCGCAT | GTTCTTGCCCATCAGCACC |  |
| *Actb* | GCTCTGGCTCCTAGCACCAT | GCCACCGATCCACACAGAGT | ATCAAGATCATTGCTCCTCCTGAGCGC |
| *Adgre1* | CAGATACAGCAATGCCAAGCA | GATTGTGAAGGTAGCATTCACAAGTG |  |
| *Adrb1* | TATCGAATCATCCGAGACGTACAG | TCCCAACTCCTCCTAAACTTTCC |  |
| *Adrb2* | TGGTGGTGATGGTCTTTGTC | GTCTTGAGGGCTTTGTGCTC |  |
| *Adrb3* | CCAGCCAGCCCTGTTGA | GGACGCGCACCTTCATAGC |  |
| *Arg1* | CTCCAAGCCAAAGTCCTTAGAG | AGGAGCTGTCATTAGGGACATC |  |
| *Cd36* | GCCAAGCTATTGCGACATGA | TCTCAATGTCCGAGACTTTTCA | CACAGACGCAGCCTCCTTTCC |
| *Chil3* | AGAAGGGAGTTTCAAACCTGGT | GTCTTGCTCATGTGTGTAAGTGA |  |
| *Cidea* | GTGGACACAGAGGAGTTCTTT | GTCGAAGGTGACTCTGGCTATTC | ACAGAAATGGACACCGGG |
| *Clec10a* | AGGTCCCTGTCATGCTTCTG | GCTGCTGGTGATCCTCTTGT |  |
| *Dgat1* | AGGTTCTCTAAAAATAACCTTGCATT | TCGTGGTATCCTGAATTGGTG |  |
| *Elovl3* | AAGGTTGTTGAACTGGGACGAC | GTGGTGGTACCAGTGGACAAA |  |
| *Fabp4* | CACCGCAGACGACAGGAAG | GCACCTGCACCAGGGC | TGAAGAGCATCATAACCCTAGATGGCGG |
| *Il10* | CAGAGCCACATGCTCCTAGA | TGTCCAGCTGGTCCTTTGTT |  |
| *Il1b* | TGGGCCTCAAAGGAAAGAAT | CAGGCTTGTGCTCTGCTTGT |  |
| *Il6* | ACACATGTTCTCTGGGAAATCGT | AAGTGCATCATCGTTGTTCATACA |  |
| *Itgam* | CAGACAGGAAGTAGCAGCTCCT | CTGGTCATGTTGATGAAGGTGCT |  |
| *Klf4* | CGGGAAGGGAGAAGACACT | GAGTTCCTCACGCCAACG |  |
| *Lipe* | GGAGCACTACAAACGCAACGA | TCGGCCACCGGTAAAGAG | CAGGCCTCAGTGTGACCGCCAGTT |
| *Lpl* | TGGAGAAGCCATCCGTGTG | TCATGCGAGCACTTCACCAG | TGCAGAGAGAGGACTCGGAGACGTGG |
| *Mrc1* | GCATGGGTTTTACTGCTACTTGATT | CAGGAATGCTTGTTCATATCTGTCTT |  |
| *Plin2* | TCATCCAGAAGCTGGAGCCA | GCAGTCTTTCCTCCATCCTGTC |  |
| *Pnpla2* | CGCCTCTCGAAGGCTCTCT | TGTAGCCCTGTTTGCACATCTC |  |
| *Ppargc1a* | AACCACACCCACAGGATCAGA | CTCTTCGCTTTATTGCTCCATGA | CAAACCCTGCCATTGTTAAGACCGAGAA |
| *Retnla* | GGGATGACTGCTACTGGGTG | TCAACGAGTAAGCACAGGCA |  |
| *Slc27a1* | CGTTTCGATGGTTATGTTAGTGACA | CATCACTAGCACGTCACCTGAGA |  |
| *Slc2a1* | GACCCTGCACCTCATTGG | GATGCTCAGATAGGACATCCAAG |  |
| *Slc2a4* | ACTCATTCTTGGACGGTTCCTC | CACCCCGAAGATGAGTGGG | TGGCGCCTACTCAGGGCTAACATCA |
| *Tbp* | CAAACCCAGAATTGTTCTCCTT | ATGTGGTCTTCCTGAATCCCT |  |
| *Tnf* | CATCTTCTCAAAATTCGAGTGACAA | TGGGAGTAGACAAGGTACAACCC | CACGTCGTAGCAAACCACCAAGTGGA |
| *Ucp1* | CCCGCTGGACACTGCC | ACCTAATGGTACTGGAAGCCTGG | AAGTCCGCCTTCAGATCCAAGGTGAAG |

**Supplementary table S1. Sequences of primers used in this manuscript.** FAM/TAMRA reporter and quencher detection system was used for genes with indicated probe sequences, and SYBR was used for the remaining genes.
